# Temporal patterns of forest seedling emergence across different disturbance histories

**DOI:** 10.1002/ece3.7568

**Published:** 2021-06-26

**Authors:** Elle J. Bowd, Lachlan McBurney, David P. Blair, David B. Lindenmayer

**Affiliations:** ^1^ Fenner School of Environment and Society College of Science The Australian National University Canberra ACT Australia

**Keywords:** fire, forests, germination, logging, post‐disturbance, recovery, seedling

## Abstract

1. Forest ecosystems experience a myriad of natural and anthropogenic disturbances that shape ecological communities. Seedling emergence is a critical, preliminary stage in the recovery of forests post​ disturbance and is triggered by a series of abiotic and biotic changes. However, the long‐term influence of different disturbance histories on patterns of seedling emergence is poorly understood.
2. Here, we address this research gap by using an 11‐year dataset gathered between 2009 and 2020 to quantify the influence of different histories of natural (wildfire) and anthropogenic (clearcut and postfire salvage logging) disturbances on emerging seedlings in early‐successional Mountain Ash forests in southeastern Australia. We also describe patterns of seedling emergence across older successional forests varying in stand age (stands that regenerated in <1900s, 1939, 1970–90, and 2007–11).
3. Seedling emergence was highest in the first three years post disturbance. Stand age and disturbance history significantly influenced the composition and abundance of plant seedlings. Specifically, in salvage‐logged forests, plant seedlings were the most different from similarly aged forests with other disturbance histories. For instance, relative to clearcut and unlogged, burnt forests of the same age, salvage logging had the lowest overall richness, the lowest counts of *Acacia* seedlings, and an absence of common species including *Acacia obliquinervia, Acacia frigescens, Cassinia arcuealta, Olearia argophylla, Pimelea axiflora, Polyscias sambucifolia,* and *Prosanthera melissifolia* over the survey period.
4. *Synthesis:* Our findings provide important new insights into the influence of different disturbance histories on regenerating forests and can help predict plant community responses to future disturbances, which may influence forest recovery under altered disturbance regimes.

## Graphical abstract

Seedling emergence is a critical, preliminary stage in the recovery of forests post disturbance. Here, using an 11‐year dataset, we provide evidence that stand age and disturbance history significantly influence the composition, richness, and abundance of plant seedlings in Mountain Ash forests in southeastern Australia across a multidecadal chronosequence.

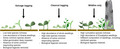

## 1 INTRODUCTION

Large, natural, stand‐replacing disturbances, including wildfire, are key drivers of the structure and composition of forest plant communities (Leverkus et al., 2018; Swanson et al., 2011; Thom & Seidl, 2016). In fire‐prone ecosystems, plant species rapidly resprout from heat‐resistant subterranean propagules and epicormic buds (Clarke et al., 2013; Lawes & Clarke, 2011) and germinate from soil seed banks (Clarke et al., 2009; Greene et al., 1999; Parrotta, 1993) or from canopy‐stored seed (serotiny) following wildfire (Ashton, 1981; Clarke et al., 2009). Indeed, wildfires are critical to the persistence of some plant species, including serotinous obligate seeders which senesce in the absence of fire (Clarke et al., 2010). In the current period of rapid environmental change, widespread anthropogenic disturbances, climatic changes, and subsequent increases in the frequency of wildfires now characterize disturbance patterns across temperate and boreal forests (Bradstock et al., 2009; Jolly et al., 2015; Sommerfeld et al., 2018; Taylor et al., 2014). Novel disturbance regimes can produce environmental conditions that exceed the adaptive resistance (ability to withstand disturbance) and resilience (ability to recover from disturbance) of plant communities. This can impede their recovery post disturbance (Auld & Denham, 2006; Enright et al., 2015; Fairman et al., 2019; Turner et al., 2019).

The emergence of seedlings post​ disturbance is a critical preliminary stage in the recovery of forest ecosystems and is triggered by several environmental and species‐specific factors (Bell, 1999; Clarke et al., 2009; Ford & HilleRisLambers, 2020; Long et al., 2015; Walck et al., 2011; Wright et al., 2018). These include the following: (a) heat and smoke produced during wildfires (that can break seed dormancy) (Auld & Denham, 2006; Flematti et al., 2004; Long et al., 2015), (b) an increase in the availability of nutrients (Chambers & Attiwill, 1994), (c) the presence of soil symbionts (Jumpponen et al., 2012) or pathogens (Ashton & Chinner, 1999), (d) the presence of remnant vegetation (Kara & Topaçoğlu, 2018), and (e) climatic conditions such as an increase in solar radiation, water availability (Ashton & Kelliher, 1996; Bell, 1994; Harper et al., 1965; Titus & del Moral, 1998), and temperature (Ford & HilleRisLambers, 2020). The composition of emerging plant species post disturbance is also regulated by the distribution, longevity, and dormancy of reproductive propagules (Palmer et al., 2018) and dispersal mechanisms or barriers of dispersal such as habitat fragmentation or distance to source population, which may be species‐specific (Primack & Miao, 1992; Tautenhahn et al., 2016).

Although disturbances produce environmental conditions that both trigger the germination of and then support emerging seedlings, increases in the intensity and frequency of disturbances and climatic changes can alter the composition and density of germinating seedlings and subsequently impact forest recovery (Donato et al., 2006; Leverkus et al., 2016; Walck et al., 2011). For instance, predicted increases in summer climatic conditions can increase the mortality of some seedlings (Marod et al., 2002), and both accelerate the release of seed dormancy and impair the resilience of soil seed banks (Ooi et al., 2009; Walck et al., 2011). Frequent disturbances also can modify environmental conditions and deplete propagule stores, resulting in a lower abundance of some plant species (Auld & Denham, 2006; Johnstone et al., 2016; Turner et al., 2019). Moreover, different disturbance origins (wildfire and clearcut and postfire salvage logging) can influence post disturbance regeneration patterns. For instance, anthropogenic disturbances such as clearcut and postfire salvage logging may have adverse impacts on the diversity of regenerating forests that differ from those of wildfire. This is because these disturbances typically involve the high‐intensity combination of mechanical clearing and postfire “slash” burning, which can alter soil properties and subsequently the soil seed bank of some species (Donato et al., 2006; Parro et al., 2015; Stark et al., 2006). Understanding the factors which influence the persistence of plant communities post disturbance is important for predicting their relative responses to future altered disturbance regimes (Palmer et al., 2018).

Previous research has described how forests can regenerate under varying intensities of single disturbances such as fire (Brown & Wu, 2005; Kennard et al., 2002), slash and burn agriculture (Miller & Kauffman, 1998), and climatic changes (Brown & Wu, 2005; Marod et al., 2002). However, understanding of the influence of prior disturbance histories, varying in origin (wildfire and logging), on early‐successional forest regeneration is limited. Further, patterns of long‐term seedling emergence in older successional forests have been poorly described in the absence of significant disturbance, which impedes understanding of forest succession. Moreover, seedling emergence research is typically conducted in laboratory‐based experiments. While these methods have been pivotal in determining the influence of future climatic changes on plant populations (Hoyle et al., 2013; Walck et al., 2011), they may underestimate the rates of germination of some species that have specific requirements to break dormancy, like natural wildfire (Baker et al., 2005; Tormo et al., 2014).

We conducted a long‐term, landscape‐scale, empirical study to assess the patterns of natural seedling emergence in the forests of southeastern Australia. We quantified seedling emergence in major shrubs as well as *Acacia* spp.*, Eucalyptus* spp. and other tree lifeforms across a multicentury chronosequence of forest ages, and in early‐successional forests with different disturbance histories. We used an extensive dataset of 1,552 observations collected over a 11‐year period to identify factors that influence in situ seedling emergence and address two important research questions: (a) What are the patterns of seedling emergence across a multicentury chronosequence. And (b) How do different disturbance histories influence seedling emergence in early‐successional forests?

At the outset of this investigation, we made four predictions about the influence of disturbance history and stand age on emerging plant seedlings.

### 1.1 Prediction #1: Higher seedling abundance in early‐successional stands, relative to older stands

We predicted that seedlings would be most abundant in young, early‐successional stands and decrease with time since disturbance. We also predicted that the composition of seedlings in young (that originated from disturbance between 2007 and 2011) stands would differ from those in intermediate (1960–70s), mature (1939), and old‐growth stands (<1900). It is well known that plant species in fire‐prone landscapes regenerate rapidly post disturbance from soil stored seed banks (Clarke et al., 2009; Greene et al., 1999; Parrotta, 1993) or canopy‐stored seed (Ashton, 1981; Clarke et al., 2009) in response to environmental cues. Seedling emergence typically declines with time since disturbance as competition increases and the availability of water, light, and nutrients decrease (Smith et al., 2014; Walck et al., 2011). However, seedling emergence of species with typically long‐lived seed banks (e.g., *Acacia* species) can occur in older forests in response to increases in light penetration and heat, or soil disturbance from fallen trees or foraging animals, although the extent that this occurs is poorly understood (Ashton & Chinner, 1999; Kara & Topaçoğlu, 2018; Strydom et al., 2017).

### 1.2 Prediction #2: Disturbance history effects on seedling abundance in early‐successional stands

We predicted that disturbance history would influence the abundance of seedlings in early‐successional forests. Specifically, we predicted that forests that were older at the time of stand‐replacing disturbance would have a higher number of emerging seedlings than forests that were younger at the time of additional disturbance. This is because forest plant species including *Acacia* and *Eucalyptus* species can have long‐lived seed stores that increase with age (Burrows et al., 2018; Leck et al., 1989; Passos et al., 2017; Strydom et al., 2017; Wang, 1997) and produce densely stocked stands that increase in density with increasing fire‐return intervals (Smith et al., 2014). Moreover, plants can dedicate more resources to reproduction as they increase in size and age (Wenk & Falster, 2015).

### 1.3 Prediction #3: Disturbance history effects on seedling composition in early‐successional stands

We predicted that disturbance history would influence the composition of emerging seedlings in early‐successional forests. Specifically, we predicted that highly disturbed forests, including those that were subject to postfire salvage logging, would have a lower abundance and a different composition of plant seedlings, lacking in diversity, relative to other early‐successional forests. This is because these forests have experienced two high‐intensity disturbances in rapid succession (natural wildfire and mechanical disturbance from logging), which can exhaust reproductive propagules and destroy natural regeneration (Blair et al., 2016). Further, salvage logging can have adverse effects on the availability of soil nutrients and soil moisture, which may impede sufficient regeneration of some species, especially those more sensitive species that require mesic environments (Bowd et al., 2019). Postfire salvage logging can also reduce species diversity, cover and richness and regeneration in other forest ecosystems (Leverkus et al., 2014), and compact soils which may displace seed banks (Cambi et al., 2015; Lindenmayer & Noss, 2006). Predicted increases in forest wildfires will likely result in an increase in subsequent salvage logging operations (Leverkus, Lindenmayer, et al., 2018; Lindenmayer & Noss, 2006; Lindenmayer et al., 2017). Understanding how forests recover after salvage logging relative to other disturbances is therefore critical for sustainable forest management.

### 1.4 Prediction #4: Environmental effects on seedling abundance and composition

We predicted that environmental variability in the landscape would influence the abundance of emerging seedlings. Specifically, we predicted that emerging seedlings would be more abundant in areas located on a northerly aspect, which typically receive more solar radiation (Aguilera et al., 2015; Ashton & Kelliher, 1996; Petter et al., 2015). Furthermore, because of the well‐known relationship between soil moisture and topography (Huggett & Cheeseman, 2002; Petter et al., 2015), we predicted that higher indices of topographical wetness would correlate to higher abundances of emerging seedlings.

We predicted that the basal area (BA) of overstory vegetation would have a positive influence on the abundance and diversity of emerging seedlings. This is because reproductive stores typically increase with stand age and the respective BA and density of overstory plants (Burrows et al., 2018; Kara & Topaçoğlu, 2018; Passos et al., 2017; Strydom et al., 2017).

## 2 METHODS

### 2.1 Study area

**FIGURE 1:**
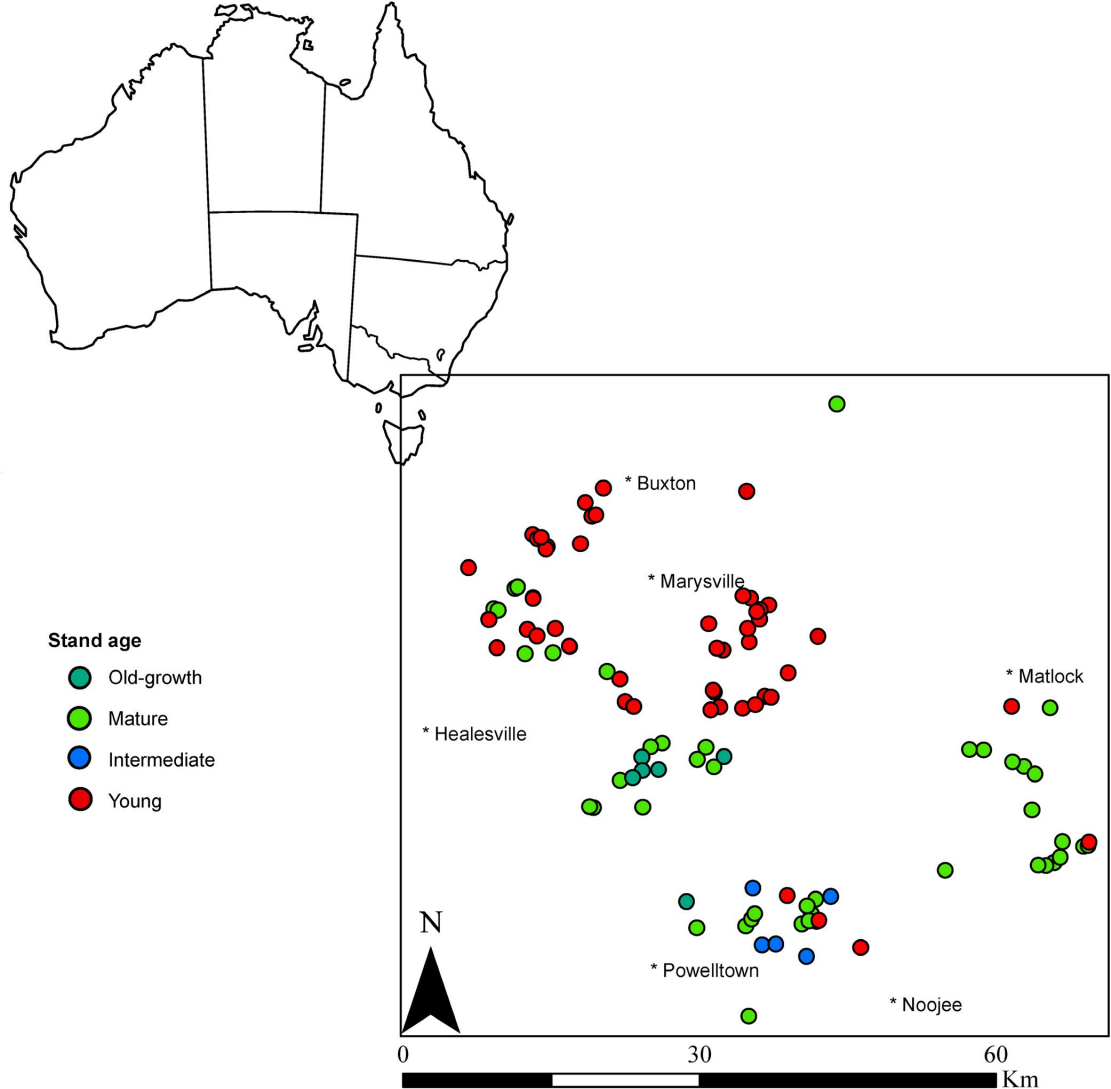
Location of 110‐survey 1‐ha sites subject to repeated sampling in the Mountain Ash forests in the Victorian Central Highlands in southeastern Australia. Colored points indicate sites with different stand ages

We conducted our study in the Mountain Ash forests of the Victorian Central Highlands, in southeastern Australia (Figure 1). These forests are dominated by the world's tallest angiosperm, *Eucalyptus regnans* (Mountain Ash), and typically occur at altitudes between 400 and 900 m (Boland et al., 2006; Costermans, 2009). They also experience high rainfall, cool winters with periods of snow, and typically mild summers. Mountain ash forest soils are primarily acidic dermosols derived from granitic rock, rich in organic matter (Bowd et al., 2019). Mean annual precipitation of this area is ~1,356.4 mm (1953–2020) and mean annual temperature ranges from a minimum of ~7.5°C to a maximum of ~15.8°C (1953–2006) (Bureau of Meteorology, 2021).

Mountain Ash forests have a rich and diverse understorey consisting of midstory trees, (including *Pomaderris aspera* and *Acacia* spp.,) broad‐leaved shrubs (such as *Olearia argophylla* and *Bedfordia arborescens*), tree ferns (*Cyathea australis* and *Dicksonia antarctica*), and a mesophilic ground layer of herbs and ferns (including *Blechnum* spp. and *Hypolepis* spp.) (Blair et al., 2016; Bowd et al., 2018).

Mountain Ash forests have a diverse and extensive history of natural and anthropogenic disturbances that have resulted in a mosaic of stand ages (Lindenmayer et al., 2019). While the historical fire‐return period in these forests is 75–150 years (McCarthy et al., 1999), in recent decades the frequency of large, high‐severity wildfires (that consume canopies and are stand‐replacing) has increased. Specifically, these forests have experienced major high‐severity wildfires in 1939, 1983, 2009, 2014, and most recently in 2019. Mountain Ash forests also have been subject to extensive clearcut logging since the 1970s and postfire (salvage) logging since the 1940s (Florence, 1996; Lindenmayer & Ough, 2006; Noble, 1977). Clearcut logging can be described as the process when all merchantable trees are removed from 15 to 45 ha cutblocks with remaining debris then burnt before a new stand of overstorey trees is regenerated using artificial reseeding. Salvage (postfire) logging occurs immediately following high‐severity wildfire and typically follows the same practices of clearcut logging of clearing, burning debris, and reseeding (Florence, 1996; Lindenmayer & Ough, 2006; Noble, 1977). As salvage logging involves two high‐intensity disturbances (wildfire and clearcut logging) in close succession, the relative effects on ecosystems are compounded (Leverkus, Rey Benayas, et al., 2018). For instance, the initial regeneration of plant species postfire is destroyed by subsequent salvage logging operations. This may exhaust reproductive plant stores and impede the extent and diversity of regenerating forests (Blair et al., 2016).

### 2.2 Experimental design

**FIGURE 2:**
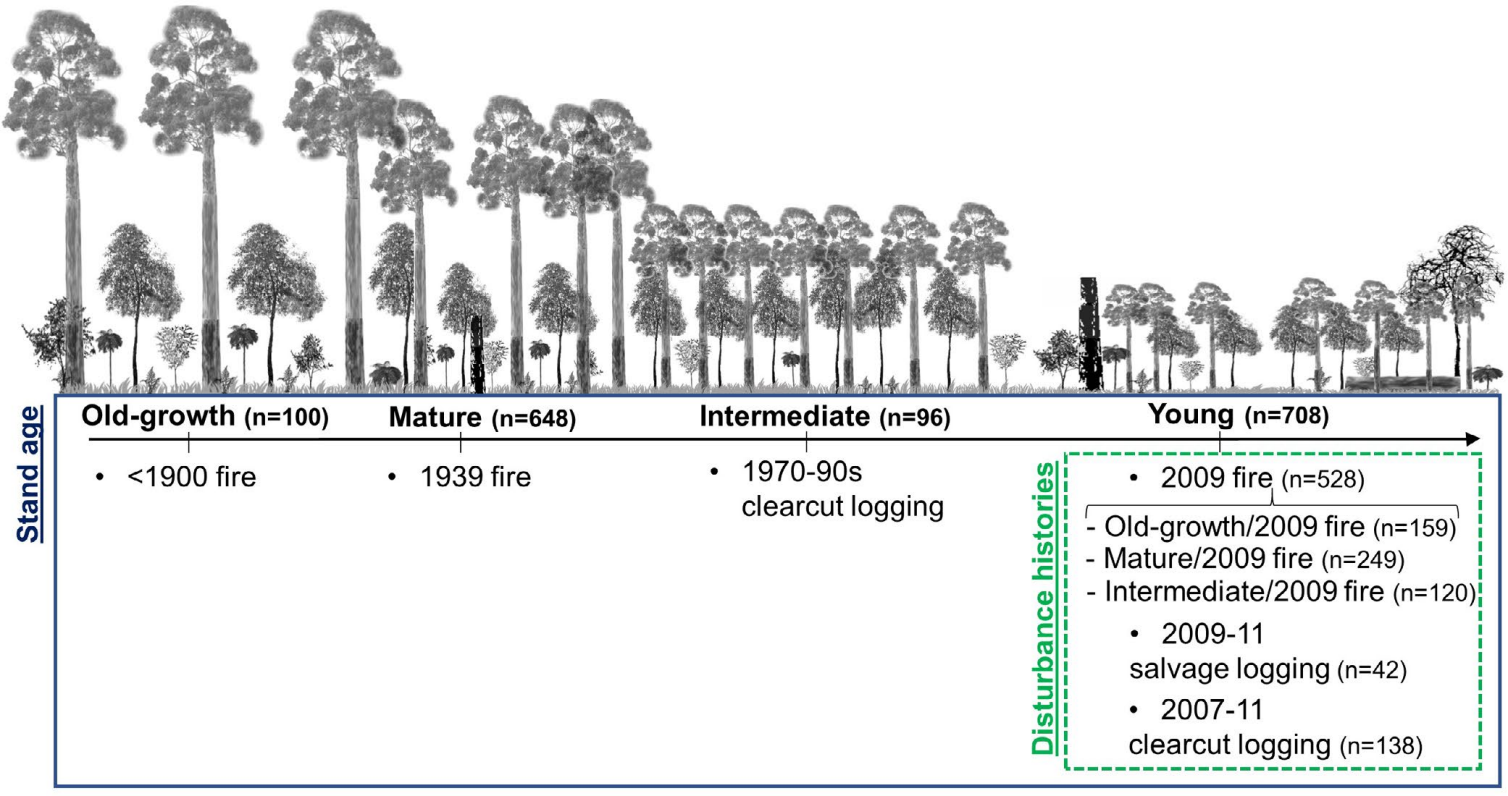
Schematic diagram detailing the experimental design and the respective stand age and disturbance history of sites. “*n*=” refers to the number of observations for each stand age and disturbance history across all survey years. Blue borders indicate observations used to quantify the influence of stand age on seedlings, and green dashed borders highlight observations used to quantify the influence of disturbance histories on seedlings in young forests only

Our survey sites were 1 ha in size and spanned a wide range of environmental conditions including stand age, slope, topographic wetness index (TWI), aspect, and disturbance history. We focused on forests subject to stand‐replacing disturbances between <1900 and 2011. These were forests that were “old‐growth” (last disturbed prior to 1900 (*n* = 100 total observations (total number of plots surveyed across all survey years))), “mature” (1939 wildfire regrowth (*n* = 648 total observations)), “intermediate” (1970–1990 logging regrowth (*n* = 96 total observations)), and early‐successional “young” stands (2007–2011 regrowth). Early‐successional “young” stands included forests that were salvage logged in 2009–11 (*n* = 42 total observations), clearcut logged in 2007–11 (*n* = 138 total observations) or those that were burnt by high‐severity wildfire in 2009 (*n* = 528 total observations). Sites that were burnt by high‐severity wildfire in 2009 also had different prior disturbance histories, including those that were previously “intermediate” aged stands logged in 1970–1990 (Intermediate/2009F) (*n* = 120 total observations), “mature” aged stands (Mature/2009F) (*n* = 249 total observations), or previously old‐growth (>50% old‐growth stands)(OG/2009F) (*n* = 159 total observations) at the time of high‐severity wildfire in 2009 (Figure 2). We used a combination of mapped information and stand‐level on‐site assessment to determine the age of stands and their relative disturbance history (Ashton, 1976).

### 2.3 Data collection

#### 2.3.1 Seedling data

We counted emerging tree, shrub, *Acacia,* and *Eucalyptus* seedlings in 367 (1 m^2^) plots across 110 (1 ha) survey sites from 2009 to 2020 (1,552 total survey observations (total number of plots surveyed across all survey years)). Specifically, in each site, we identified, counted, and measured the height of seedlings in three 1 × 1 m plots, 10, 50, and 90 m along a 100 m central transect. We visited >80% of survey sites between three and seven times across the 11‐year sampling period to document longitudinal trends in seedling emergence. We excluded plots from the analysis if they were extensively damaged by large fallen branches or trees. In our study, we included only shrub seedlings <50 cm in height and *Acacia, Eucalyptus,* and other tree species <200 cm in height to account for different growth forms and growth rates. We did not include exotic species in our analyses, which are rare in Mountain Ash forests (Blair et al., 2016). Moreover, emerging seedlings documented in this study had germinated from the soil seed bank and did not include resprouts from fire‐killed plants or subterranean organs.

#### 2.3.2 Overstorey basal area

From the same 110 sites described above, we concurrently measured the diameter at breast height (DBH) of *Acacia, Eucalyptus*, tree, and shrub species greater than 2 m in height across three 10 × 10 m quadrats located 10, 50, and 90 m along a central 100 m transect. Using pooled DBH measures across quadrats, we calculated the Basal Area (BA) of *Acacia, Eucalyptus*, tree, and shrub species at the site level (m^2^/ha). BA measures can fluctuate with time since disturbance as live tress perish and as new regrowth vegetation develops.

### 2.4 Statistical analysis

We modeled the count (abundance) of emerging tree, *Eucalyptus* and *Acacia* seedlings (<200 cm), and shrub seedlings (<50 cm) using two sets of Bayesian regression models. We generated the first set of models using all 1,552 observations across all stand ages, and the second set of models using early‐successional sites that regenerated in 2007–11 only, controlling for the influence of stand age (708 observations total). The hurdle component of both model sets was used to account for the high number of zero counts of tree*, Eucalyptus*, *Acacia*, and shrub seedlings. For the conditional component of both model sets, we used a truncated negative binomial distribution to account for overdispersion. For both model sets, we performed model selection on each component (conditional and hurdle) of the model independently.

Specifically, for the first model set, we fit all combinations of six covariates: stand age (categorical variable), TWI (scaled numeric variable), slope (scaled numeric variable), N aspect (categorical variable), survey year (scaled numerical variable), and the mean BA of the respective lifeform (*Acacia, Eucalyptus*, shrub, tree) (scaled numeric variable), with random effects: site or site/plot number. For each lifeform, we chose the model that included stand age in both model components with the lowest Widely Applicable Information Criterion (WAIC) (Gelman & Rubin, 1992; Vehtari et al., 2017). Specifically, the model was as follows:

$${\text{SC}}_{i}∼\text{HNB}\left(\mu_{i},\theta \right)$$

$$\mu_{i}=\text{Intercept}+{\text{SurveyYear}}_{i}+{\text{TWI}}_{i}+{\text{Slope}}_{i}+{\text{NortherlyAspect}}_{i}+{\text{StandAge}}_{i}+{\text{LifeformBA}}_{i}+{\text{SiteCodeNB}}_{i}$$

$${\text{HU}}_{i}=\text{Intercept}+{\text{SurveyYear}}_{i}+{\text{TWI}}_{i}+{\text{Slope}}_{i}+{\text{NortherlyAspect}}_{i}+{\text{StandAge}}_{i}+{\text{LifeformBA}}_{i}+{\text{SiteCodeHU}}_{i}$$

where the covariates are the same as previously described, SC is the seedling count on the *i*th site, HNB is the hurdle negative binomial, HU*_i_* is the hurdle model component, *μ_i_* is the mean of the negative binomial distribution and *θ* is the shape parameter of the negative binomial distribution. Random effects are SiteCode_NB_ for the negative binomial component of the model and SiteCode_HU_ for the hurdle component of the model. We modified random effects to either SiteCode_HU_ or SiteCode/PlotNumber_HU_ for the hurdle model component, and SiteCode_NB_ or SiteCode/PlotNumber_NB_ for the negative binomial model component based on model selection. The log link was used for the negative binomial component of each model, and the logit link for the hurdle component of each model.

For the second set of models, we fit all combinations of six covariates: time since disturbance (scaled numeric variable), TWI (scaled numeric variable), slope (scaled numeric variable), N aspect (categorical variable), the mean BA overstory of the respective lifeform (*Acacia, Eucalyptus*, shrub, tree) (scaled numeric variable), and disturbance history (categorical variable), with random effects: site or site/plot number. We chose the model that included disturbance history in both model components with the lowest Widely Applicable Information Criterion (WAIC) criterion (Gelman & Rubin, 1992; Vehtari et al., 2017). These models followed the same structure as the first model subset, but had different parameters. Specifically, the model was as follows:

$${\text{SC}}_{i}∼\text{HNB}\left(\mu_{i},\theta \right).$$

$$\mu_{i}=\text{Intercept}+{\text{TimeSinceDisturbance}}_{i}+{\text{TWI}}_{i}+{\text{Slope}}_{i}+{\text{NortherlyAspect}}_{i}+{\text{DisturbanceHistory}}_{i}+{\text{LifeformBA}}_{i}+{\text{SiteCodeNB}}_{i}$$

$${\text{HU}}_{i}=\text{Intercept}+{\text{TimeSinceDisturbance}}_{i}+{\text{TWI}}_{i}+{\text{Slope}}_{i}+{\text{NortherlyAspect}}_{i}+{\text{DisturbanceHistory}}_{i}+{\text{LifeformBA}}_{i}+{\text{SiteCodeHU}}_{i}$$

where the covariates are the same as previously described, SC is the seedling count on the *i*th site, HNB is the hurdle negative binomial, HU*_i_* is the hurdle model component, *μ_i_* is the mean of the negative binomial distribution, and *θ* is the shape parameter of the negative binomial distribution. Random effects are SiteCode_NB_ for the negative binomial component of the model and SiteCode_HU_ for the hurdle component of the model. We modified random effects to either SiteCode_HU_ or SiteCode/PlotNumber_HU_ for the hurdle model component, and SiteCode_NB_ or SiteCode/PlotNumber_NB_ for the negative binomial model component based on model selection. The log link was used for the negative binomial component of each model, and the logit link for the hurdle component of each model.

We fit all models using the brms (Bayesian regression models using Stan) package in R (Buerkner, 2017). We used student‐t priors with eight degrees of freedom, zero mean and scale parameter of 1.5 for the regression parameters for the hurdle and negative binomial model components and used defaults student‐t priors with three degrees of freedom, zero mean and scale parameter of 2.5 for the standard deviation of random effects for both model components (Gelman et al., 2008).

We ran four Markov chains for 4,000 iterations, discarding the first 2,000 as warm‐up leaving 4,000 posterior samples for inference and applied a thinning parameter of two. We assessed model convergence using the $\hat{R}$ statistic (Gelman & Rubin, 1992). All model $\hat{R}$ statistics were less than 1.01 indicating adequate mixing of the chains. We report posterior estimates, means and 95% credible intervals. All analyses were conducted in R version 4.0.2 (R Core Team, 2019). A single outlier observation of >1,600 seedlings in a single 1 × 1 m plot was removed from all models.

Using the mean count of individual tree, *Eucalyptus* spp., *Acacia* and shrub seedlings for each site across all years, we determined the influence of stand age, TWI, slope, northerly aspect, and the mean BA of overstorey *Eucalyptus*, tree, shrub, and *Acacia* lifeforms on the overall composition of emerging seedlings using permutational multivariate analysis of variance (PERMANOVA) based on a Bray‐Curtis dissimilarity matrix of square‐root transformed data using the “*adonis*” and “*vegdist*” functions in the “*vegan”* package in R (Dixon, 2003). We used the same method for the mean count of individual tree, *Eucalyptus,* *Acacia* and shrub seedlings in early‐successional sites within the first three years of disturbance to determine the influence of disturbance history, TWI, slope, and northerly aspect on the composition of emerging seedlings in these forests, controlling for stand age.

For both data sets, we conducted pairwise testing between different stand ages, and disturbance histories in early‐successional forests to examine the difference in the composition of seedlings using PERMANOVA based on a Bray‐Curtis dissimilarity matrix of square‐root transformed data using the “pairwiseAdonis” and “vegan” packages in R (Martinez Arbizu, 2020; Dixon, 2003). For pairwise comparisons, we adjusted p‐values using “Bonferroni” corrections to account for potential type I errors. We removed sites from multivariate analyses that had zero seedlings based on the requirements of the analysis. In total, we removed one site from the early‐successional data subset, and 10 sites from the stand age data subset. In the early‐successional dataset, we used the mean count of seedlings within the first 3 years of disturbance for multivariate analyses because this is the period when seedlings were most abundant as indicated by Bayesian regression models.

## 3 RESULTS

We counted a total of 9,750 tree, shrub, *Eucalyptus,* and *Acacia* seedlings across the 110 sites during the 11 year sampling period (1,552 observations). *Eucalyptus* seedlings (3,862 individuals) and *Acacia* (3,136 individuals) seedlings were the most abundant, followed by tree seedlings (1,506 individuals) and shrub seedlings (1,246 individuals). Across all sites, we identified 49 different species of emerging seedlings: seven *Acacia* species, four *Eucalyptus* species, 28 shrub species and 10 tree species (Table A1).

### 3.1 Seedling emergence across a multicentury chronosequence

#### 3.1.1 Seedling richness

**FIGURE 3:**
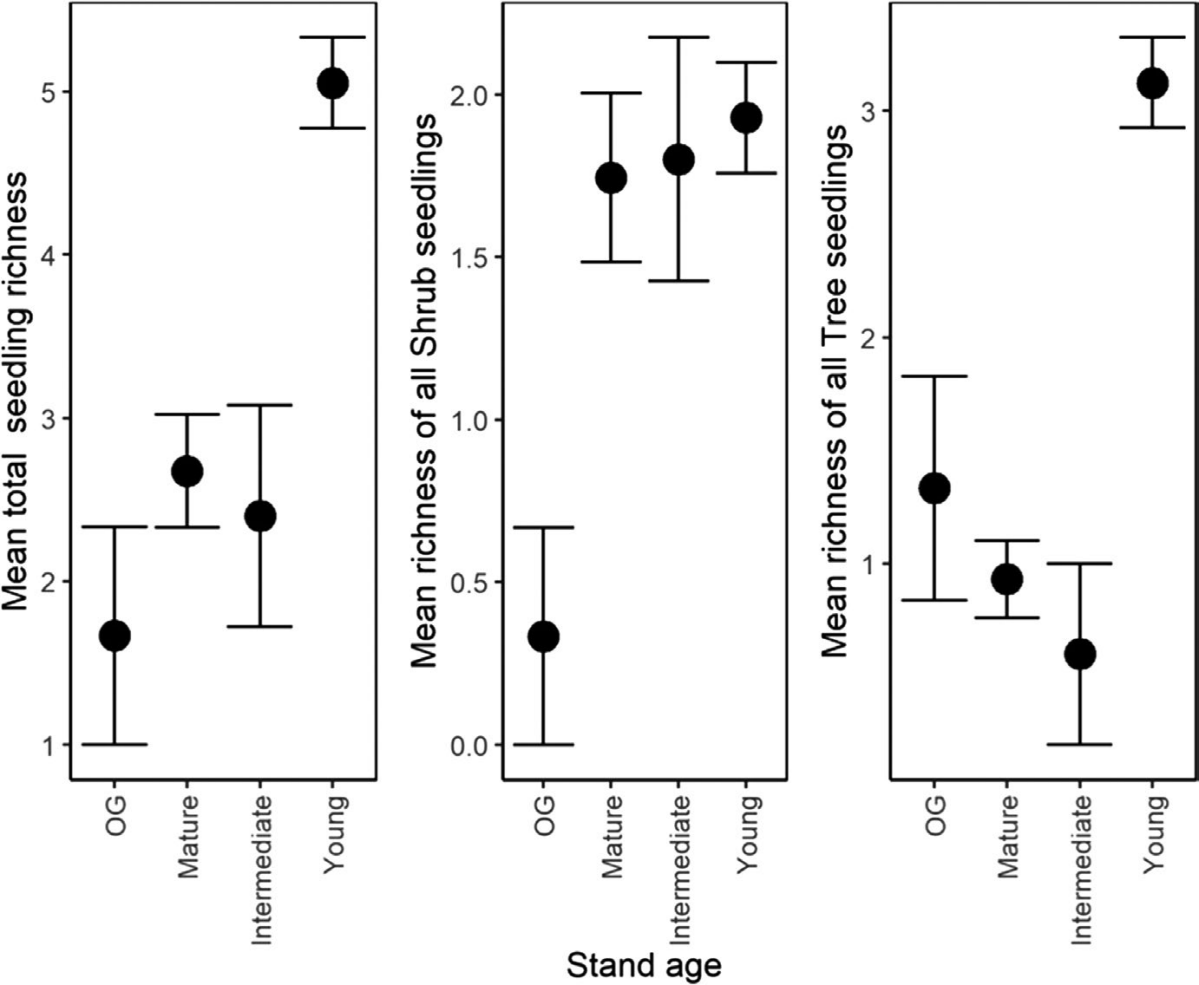
Mean richness of total, shrub, and tree (inclusive of *Acacia* and *Eucalyptus*) seedlings for each stand age

Pooling across survey years, the mean total richness of all seedlings and shrub seedlings was highest in young stands (total seedling richness = 5.05 ± 0.28; shrub seedling richness = 1.93 ± 0.17), and lowest in old‐growth stands (total seedling richness = 1.67 ± 0.67; shrub seedling richness = 0.33 ± 0.33). Moreover, the mean richness of all tree lifeforms including *Acacia* and *Eucalyptus* seedlings was highest in young stands (3.12 ± 0.2), and lowest in intermediate stands (0.6 ± 0.4) (Figure 3). However, the cumulative richness of seedlings was highest in mature aged stands (37 unique species across all sites), and lowest in old‐growth and intermediate stands (seven unique species across all sites) (Table A2).

#### 3.1.2 Seedling abundance

**FIGURE A1:**
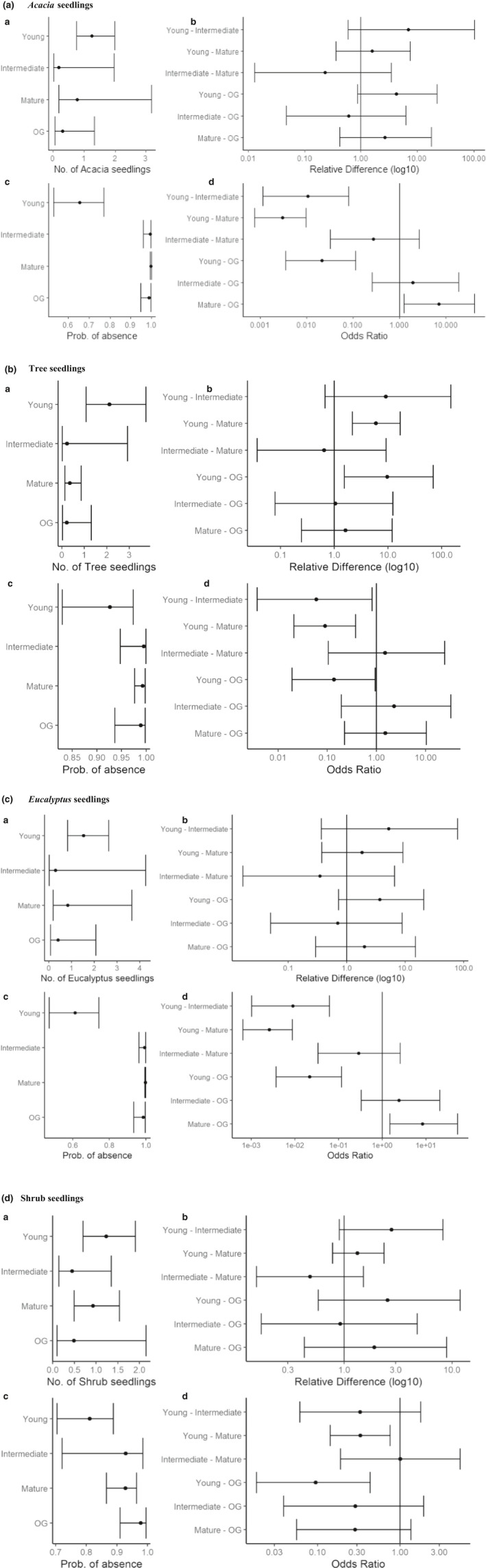
Estimates and comparisons with 95% credible intervals of the effects of stand age on the (A) conditional abundance and (B) corresponding pairwise comparisons, (C) unconditional abundance and (D) corresponding pairwise comparisons, and (E) probability of absence and (F) corresponding pairwise comparisons of *Acacia*, tree, *Eucalyptus,* and shrub seedlings

**FIGURE A2:**
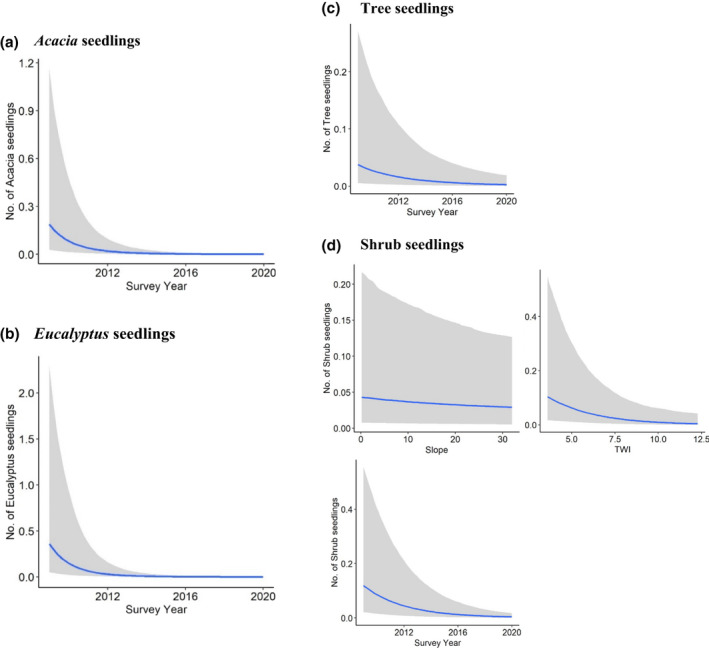
Predicted count of seedlings for environmental and temporal variables with 95% credible intervals. These predictions were generated from both truncated negative binomial and hurdle model components where applicable and are only displayed for significant environmental and temporal variables included in each respective model. Full model details are located in Table A2

**FIGURE A3:**
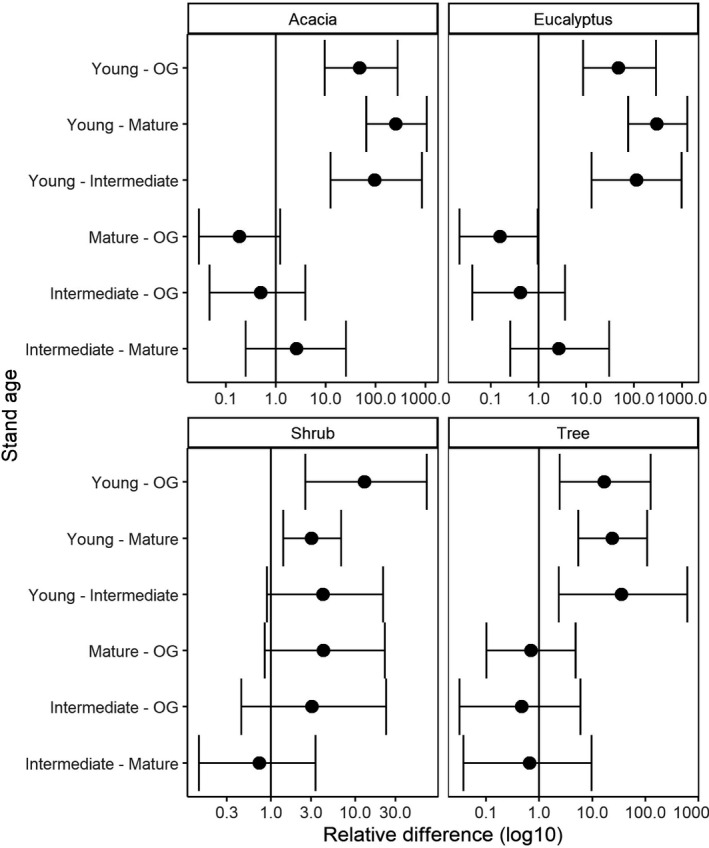
Relative differences in the mean number of seedlings between stand ages with 95% credible intervals. Mean estimates were generated from both negative truncated binomial and hurdle components of each model. Full model details are given in Table A3. Relative differences based on mean estimates from each model component independently are shown in Figure A1. OG = old‐growth

After conditioning on the presence of seedlings within a given site, we found a negative association between the number of seedlings of all plant lifeforms and survey year (Figure A1, Figure A2, Table A3), indicating that as time since disturbance increased, emerging seedlings decreased (relative 95% credible intervals (CI) of seedling estimates = Acacia [−1.68,‐1.24]; tree [−1.03,‐0.32]; *Eucalyptus* [−1.75,‐1.22]; shrub [−0.64,‐0.22]). This trend also was evident in the hurdle component of the model, where survey year was associated with a higher probability of zero seedlings across all lifeforms (CI of seedling estimates = Acacia [1.09, 1.67]; tree [0.31, 0.79]; *Eucalyptus* [1.19, 1.76]; shrub [0.53, 0.91]). Moreover, young stands were associated with a higher number of tree seedlings [0.43, 4.24], and a lower probability of zero seedlings across all lifeforms, relative to old‐growth stands (CI of seedling estimates = Acacia [−5.63,‐2.17]; tree [−3.96, −0.05]; *Eucalyptus* [−5.6,‐2.15]; shrub [−4,‐0.83]). In contrast, mature stands were associated with an increased probability of zero *Acacia* and *Eucalyptus* seedlings, relative to old‐growth stands (CI of seedling estimates = Acacia [0.22, 3.73] and *Eucalyptus* [0.4, 3.98]) (Figures A1,A3, Table A3).

We found limited evidence of the influence of environmental variables on the abundance of emerging seedlings. However, we found a positive association between indices of topographical wetness (CI of estimate = [0.11, 0.94]) and the BA of shrub species (CI of estimate = [0.06, 0.7]), and the probability of zero shrub seedlings (Figure A2, Table A3).

**FIGURE 4:**
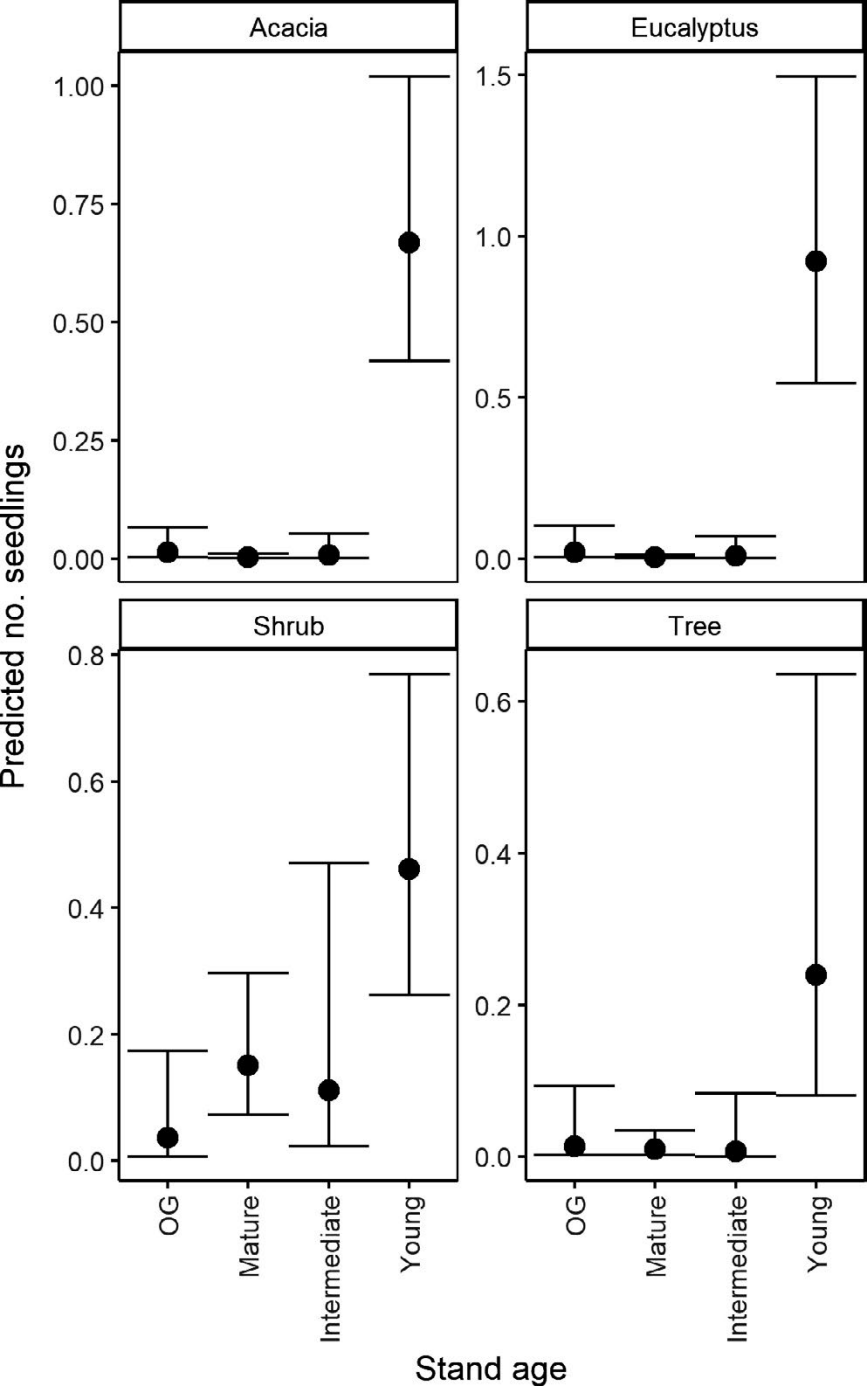
Predicted count (and 95% credible intervals) of seedlings for each stand age class. Predictions were generated from both the truncated negative binomial and hurdle model components. Full model details are in Tables A3, A4 and Figure A4. Relative comparisons are displayed in Figure A3. OG = old‐growth

Consistent with our predictions at the outset of this investigation, the predicted number of seedlings was highest in young forests regenerating from stand‐replacing disturbance in 2007–11, relative to old‐growth forests (Figure 4). Pairwise contrasts based on the relative differences between stand ages indicated that early‐successional, young forests had significantly higher predicted abundances of *Acacia,* tree, and *Eucalyptus* seedlings than all other stand ages (Figure A3). However, we also found evidence of seedling emergence in older forests outside of stand‐replacing disturbance events. For instance, old‐growth stands had a higher predicted abundance of *Eucalyptus* seedlings, than mature aged stands (Figure A3).

#### 3.1.3 Seedling community composition

Multivariate permutational analysis of variance (PERMANOVA) provided evidence that TWI and stand age influenced the composition of tree, shrub, *Eucalyptus,* and *Acacia* seedlings (Table A5). Further pairwise testing revealed significant differences in the composition of seedlings between young‐aged stands and all other stand ages (Table A5). Seedling emergence in old‐growth forests included cool temperate rainforest dominant species, *Nothofagus cunninghammii* and *Atherosperma moschatum,* which were not found in young or intermediate‐aged stands. Emerging seedlings in young, early‐successional forests included species which produce persistent on‐site seed stores, including *Prosanthera lasianthos, P. aspera, Hedycarya angustifolia, Zieria arborescens* and *Olearia* species, all which were absent from seedling plots in old‐growth forest (Table A2).

### 3.2 Seedling emergence in early‐successional forest

#### 3.2.1 Seedling richness

**FIGURE 5:**
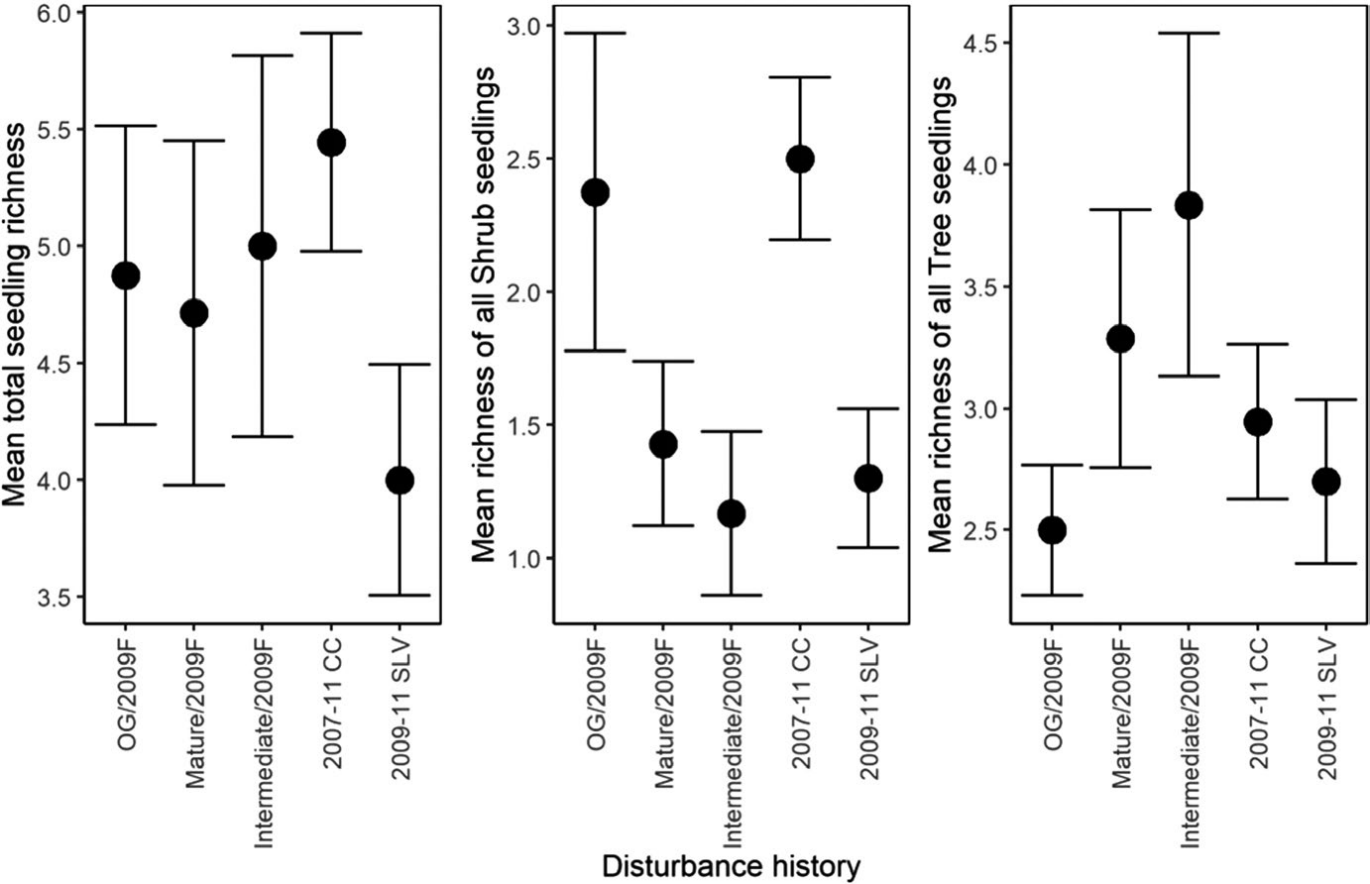
Mean total, shrub, and total tree (inclusive of *Acacia* and *Eucalyptus*) seedling richness for each disturbance history in early‐successional forests

Pooling across the first three years post disturbance, the total richness of seedlings was highest in sites that were clearcut logged in 2007–11 (5.44 ± 0.47) and lowest in salvage‐logged sites (4 ± 0.494). Mean shrub seedling richness was also highest in sites clearcut logged in 2007–11 (2.5 ± 0.31) and in prior old‐growth forests, burnt in 2009 (2.38 ± 0.6), and lowest in prior intermediate‐aged forests, burnt in 2009 (1.17 ± 0.31). In contrast, the mean richness of all tree seedlings (inclusive of *Eucalyptus* and *Acacia*), was highest in prior intermediate‐aged forests, burnt in 2009 (3.83 ± 0.7), and lowest in prior old‐growth forests, burnt in 2009 (2.5 ± 0.27) (Figure 5). Salvage‐logged sites had the lowest cumulative species richness (11 unique species across all sites), and prior mature forests, burnt in 2009 had the highest (23 unique species across all sites) (Table A6).

#### 3.2.2 Seedling abundance

**FIGURE 6:**
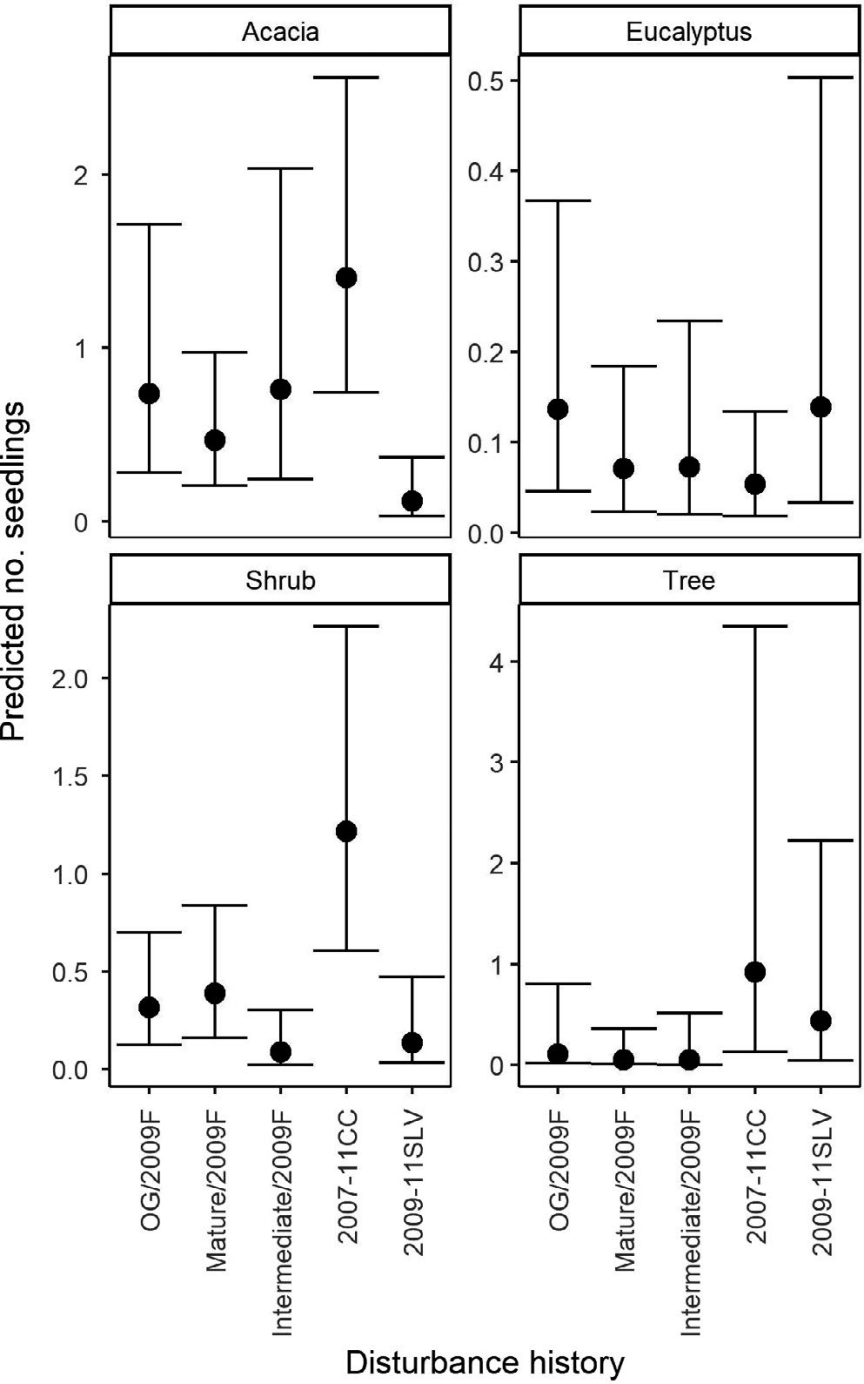
Predicted count of seedlings for each class disturbance history with 95% credible intervals. Predictions were generated from both the truncated negative binomial and hurdle model components. Full model details are located in Tables A7, A8 and Figure A8. Relative comparisons are displayed in Figure A7. OG = old‐growth

**FIGURE A5:**
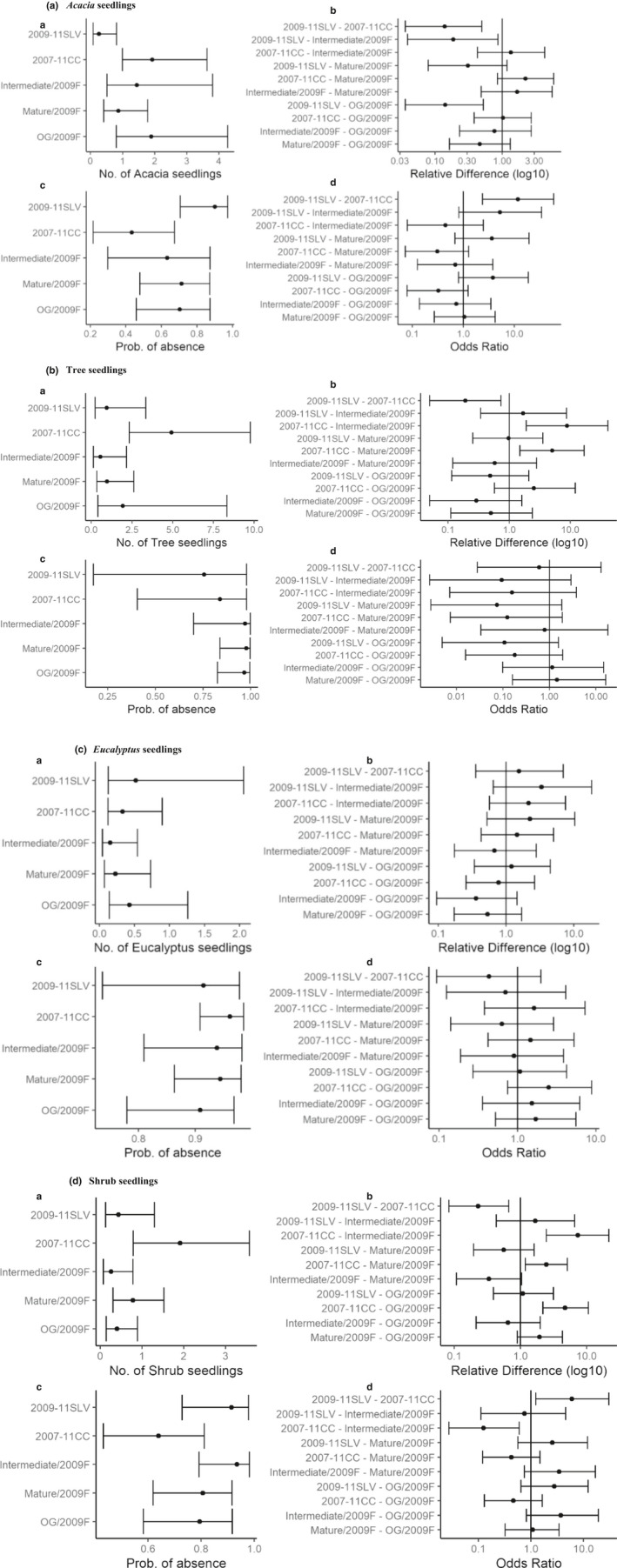
Estimates and comparisons with 95% credible intervals of the effects of disturbance history on the (A) conditional abundance and (B) corresponding pairwise comparisons, and the (C) probability of absence and (F) corresponding pairwise comparisons of *Acacia*, tree, *Eucalyptus* and shrub seedlings. “CC” = clearcut logging, “F” = fire, “SLV” = salvage logging

**FIGURE A6:**
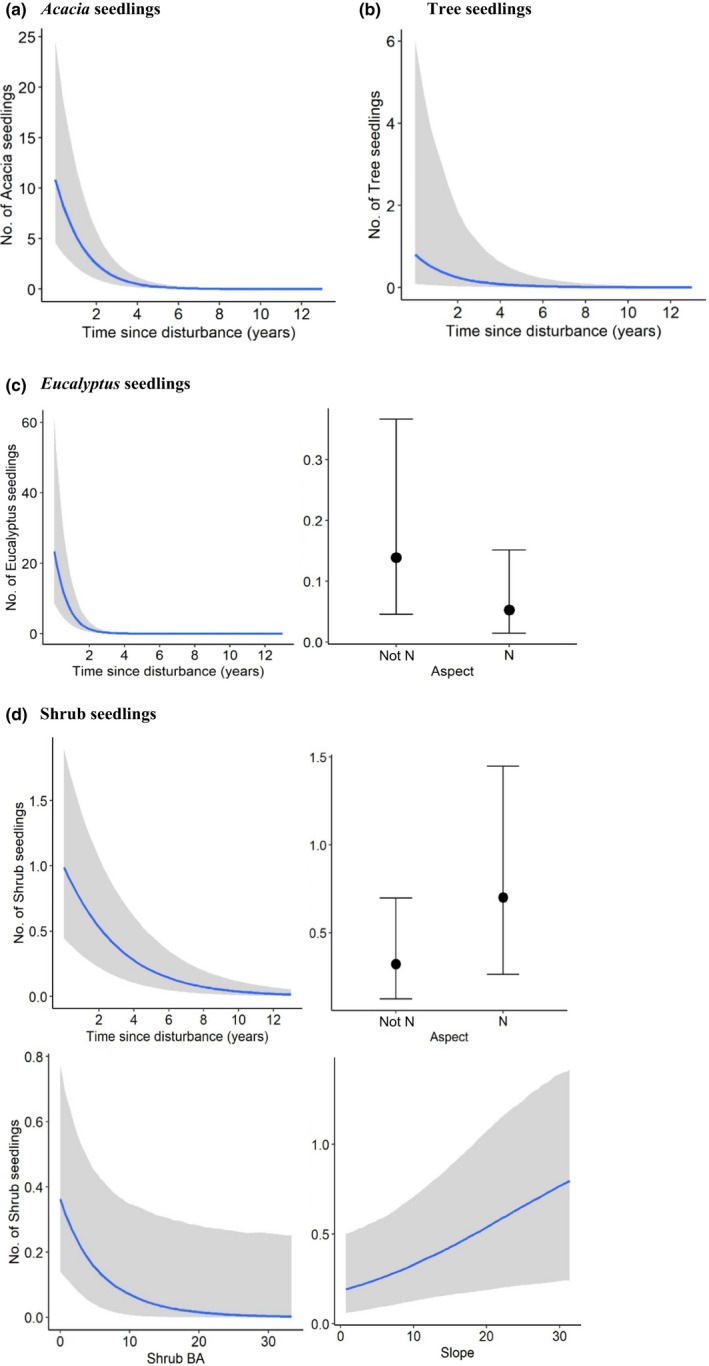
Predicted count of seedlings for environmental and temporal variables included in each model with 95% credible intervals. These predictions were generated from both the truncated negative binomial and hurdle model components where applicable and are only displayed for significant environmental and temporal variables included in each respective model. Full model details are located in Table A5

After conditioning for the presence of seedlings within a given site, we found that the numbers of *Eucalyptus*, tree, shrub, and *Acacia* seedlings declined significantly with increasing time since disturbance in early‐successional, young forests (CI of seedling estimates = Acacia [−1.78,‐1.32]; *Eucalyptus* [−3.89,‐2.44]; shrub [−1.02, −0.33]; tree [−1.41, −0.58]) (Table A7, Figures A5,A6). Specifically, seedlings of all lifeforms were most abundant in the first three years post disturbance (Figure A6). We also found evidence of an effect of disturbance history on the abundance of seedlings in these young forest stands. Relative to sites that were old‐growth prior to being burnt in 2009, salvage‐logged sites supported fewer *Acacia* seedlings (CI of estimate [−3.33, −0.65]), and clearcut logged sites supported a higher abundance of shrub seedlings (CI of estimate [0.78, 2.36]) (Figure 6, Table A7).

In these early‐successional forests, we also found a paucity of effects of environmental variables on the abundance of seedlings. However, when conditioning on the presence of seedlings, northerly aspects were associated with lower counts of *Eucalyptus* seedlings (CI of estimate [−3.17, −0.78]), and higher counts of shrub seedlings (CI of estimate [0.05, 1.29]). Moreover, steep slopes decreased the probability of zero shrub seedlings (CI of estimate [−0.96, −0.08]) and, the BA of shrub species increased the probability of zero shrub seedlings (CI of estimate [0.01, 0.92]) (Table A7, Figure A6).

**FIGURE A7:**
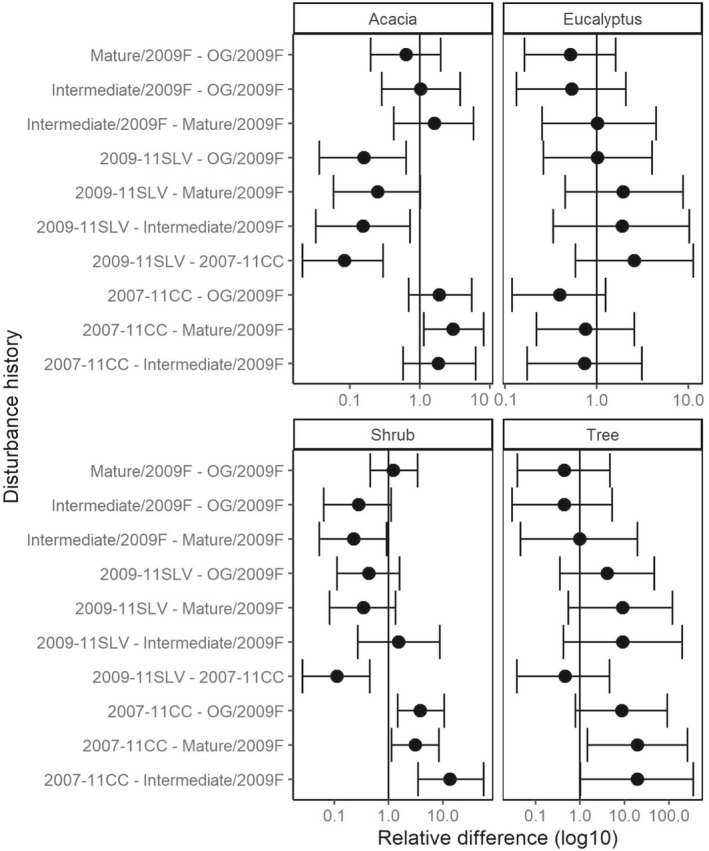
Relative differences in the mean number of seedlings between different disturbance histories in early successional forests with 95% credible intervals. Mean estimates were generated from both the negative truncated binomial and hurdle components of each model. Full model details are located in Table A6. Relative differences based on mean estimates from each model component independently are located in Figure A4

Relative pairwise contrasts indicated that clearcut logged stands had a higher predicted abundance of shrub seedlings, relative to all other early‐successional young stands (Figure A7). Salvage‐logged stands had a lower predicted abundance of *Acacia*, relative to all other early‐successional young stands (Figure A7). In contrast, sites subject to clearcut logging between 2007 and 2011 had a higher predicted abundance of *Acacia* and tree seedlings, relative to prior mature forests, burnt in 2009.

#### 3.2.3 Seedling community composition

The composition of plant seedlings significantly differed with different disturbance histories and the basal area of *Eucalyptus* spp. in early‐successional forests (Table A9). Further pairwise testing indicated that the composition of seedlings in salvage‐logged forests was different from all other disturbance histories, except for Intermediate/2009F sites, which also had been subject to compounding disturbances. These differences are likely explained by salvage‐logged stands having the lowest seedling diversity and mean abundances of some tree, shrub, and *Acacia* seedlings, relative to similarly aged sites with different disturbance histories. For instance, sites subject to salvage logging supported low overall mean abundances of *Acacia dealbata* and *E. regnans*. Moreover, species that were generally common in Mountain Ash forests such as *Acacia obliquinervia, Acacia frigescens, Cassinia arcuealta, O. argophylla, Pimelea axiflora, Sambucus gaudichaudiana*, *Polyscias sambucifolia* and *Prosanthera melissifolia* were absent from all salvage‐logged plots; however, they did occur in forests of the same age with different disturbance histories (Table A6).

The composition of seedlings in Intermediate/2009F sites that were previously clearcut logged in 1970–90 and then burnt in 2009 differed from sites that were clearcut logged in 2007–11 (Table A9). Indeed, Intermediate/2009F sites had no emergence of common shrub species such as *Cassinia aculeata, P. sambucifolia*, *Olearia lirata, P. axiflora, Z. arborescens, Coprosma quadrifida* or *Olearia phlogopappa* across all sites.

We found no evidence of compositional differences in other pairwise tests. However, common resprouting shrubs *O. argophylla* and *S. gaudichaudiana* were absent from sites that were clearcut logged in 2007–11 but occurred in other early‐successional sites that regenerated from wildfire in 2009 (Table A6).

Moreover, when pooling across seedling surveys from the first three years post disturbance, prior old‐growth forests, burnt in 2009 had the highest mean number of dominant species, *E. regnans* seedlings (28.03 ± 18.48)​ and the second highest mean abundance of common *A. dealbata* (4.7 ± 1.3) (Table A6).

## 4 DISCUSSION

Seedling emergence is a preliminary indicator of the recovery of forest ecosystems postdisturbance and is important for predicting long‐term responses to future altered disturbance regimes (Bače et al., 2015; Johnstone et al., 2004; Palmer et al., 2018; Sass et al., 2018). Several studies have described the influence of single disturbance events on early forest regeneration (Johnstone et al., 2004; Leverkus, et al., 2018). However, understanding of the long‐term patterns of seedling emergence in forests along successional gradients, and with different prior disturbance histories (varying in origin) is limited (Parro et al., 2015). Using over a decade of longitudinal data, we provide empirical evidence that different disturbance histories influence the recovery of forest plant communities in early‐successional stages. Moreover, we describe patterns of seedling emergence in forests that were last disturbed in <1900s, 1939, 1970–90s, and 2007–11.

Consistent with our first prediction, we found that the highest abundance of emerging seedlings was within the first three years postdisturbance. However, we also uncovered evidence of seedling emergence across our multicentury chronosequence. For instance, *Acacia* seedlings were the most persistent and occupied 55% of all sites surveyed, with >50% occupancy in old‐growth sites alone (during the survey period). As outlined in predictions #2 and #3, seedling emergence in early‐successional salvage‐logged forests differed from that in forests of the same age subject to different disturbance histories. Specifically, these forests had the lowest cumulative species richness of seedlings and abundance of *Acacia* seedlings, relative to unlogged, burnt, and clearcut forests of the same age. In contrast, unlogged, previously mature forests that were subsequently burnt in 2009 had the highest cumulative richness of tree, *Acacia* and *Eucalyptus* seedlings, and clearcut logged forests had the highest abundance and richness of shrub seedlings. Moreover, in contrast to prediction #4, we found a paucity of environmental influences on emerging seedlings. This suggests disturbance history and stand age are likely the dominant drivers of patterns of seedling emergence in our study area. With global disturbance patterns predicted to increase and intensify in future years, our findings provide a timely insight into the influence of different disturbance histories on regenerating forests to provide for forest management.

### 4.1 Seedling emergence across a multicentury chronosequence

Consistent with our first prediction at the outset of this investigation, and congruent with the findings of other research (Balch et al., 2013; Smith et al., 2014; Tsuyuzaki et al., 2014), we found that seedling emergence is typically highest within the first 2–5 years post‐disturbance. The relatively high abundances of *Acacia* and *Eucalyptus* seedlings in young forest stands during this period were the most divergent from other stand ages. This reflects the rapid‐regeneration responses of these species to stand‐replacing disturbances (Bowd et al., 2021) and the relative environmental conditions that stimulate and support their germination (Auld & Denham, 2006; Chambers & Attiwill, 1994; Flematti et al., 2004; Long et al., 2015).

Patterns of seedling emergence in older successional forests had different species compositions and a low overall diversity, relative to younger forests. The low diversity of emerging seedlings in older successional forests may be explained by several unmeasured factors that can limit the germination and presence of some species in the soil seed bank. These include potential spatial and temporal variation in the abundance of overstorey species, and species‐specific differences in dispersal mechanisms (Primack & Miao, 1992; Tautenhahn et al., 2016) and the longevity of reproductive propagules, which can be depleted over time through predation, pathogen attack, and declines in viability (Ashton & Chinner, 1999; Auld et al., 2000; Palmer et al., 2018). Moreover, the low diversity of seedlings in older forests is likely attributed to an absence of sufficient stimulus to trigger significant germination events (e.g., heat, solar radiation, smoke) (Auld & Denham, 2006; Flematti et al., 2004; Kara & Topaçoğlu, 2018; Long et al., 2015). However, seedling emergence in older forests can be triggered by environmental conditions including light availability and abiotic soil conditions altered by falling trees/limbs or foraging fauna (Ashton & Chinner, 1999; Kara & Topaçoğlu, 2018). For instance, the Superb Lyrebird (*Menura novaehollandiae)* can turnover up to 200 tonnes of leaf litter and soil annually, which may prompt seed germination (Ashton & Bassett, 1997). Further, seedlings of some plant species can have a high survival rate under an established overstorey canopy, although they typically grow at a slower rate than where the canopy has been removed (Dechoum et al., 2015).

Emerging seedlings in old‐growth forests included *A. dealbata, A. frigescens* and cool temperate rainforest dominants including *A. moschatum* and *Nothofagus cunninghamii*. Surprisingly, >50% of old‐growth Mountain Ash forests sites had *Acacia* seedlings present during the survey period. *Acacia* species have particularly long‐lived seed banks, which accumulate over time and remain viable for many decades (Burrows et al., 2018; Strydom et al., 2017). This allows these species to persist in ecosystems for long periods and has contributed to them being invasive species in some ecosystems (Passos et al., 2017; Strydom et al., 2017). Other recent studies have recorded declines in *Acacia* species in older successional forests (Forrester et al., 2011; Trouvé et al., 2019). However, our study provides evidence that *Acacia* species can indeed persist in older successional forests and produce viable seedlings for over 100 years. As large standing trees begin to senesce, some level of natural regeneration is important for the persistence of these plant species and others which play key functional roles in forest ecosystems including nitrogen fixation (Chaer et al., 2011; May & Attiwill, 2003) and providing habitat and a foraging substrate for mammals and birds (Broadhurst & Young, 2006; Smith, 1984; Whelan & Maina, 2005).

As seedling germination in older successional stages was limited to specific lifeforms with specific functional traits (long‐lived soil seed banks), these findings are consistent with vital attributes successional theory (Noble & Slatyer, 1980). Further, the germination of rainforest species, including *A. moschatum* and *N. cunninghamii* in later successional forests is consistent with the Initial Floristics Composition model of successional theory (Egler, 1954; Pulsford et al., 2014).

### 4.2 Disturbance history influences seedling emergence in early‐successional forests

At the outset of this study, we predicted that older forests at the time of disturbance would have a higher abundance of seedlings than forests that were younger at the time of disturbance (prediction #2). While we did not find any significant influence of prior stand age on the abundance of seedlings, forests that were old‐growth prior to being burnt in 2009 were characterized by a high mean richness of shrub species and a high mean abundance of dominant midstory species, *A. dealbata* and dominant overstorey species, *E. regnans* (Table A6). In forests regenerating from recent wildfire, a high diversity and high seedling abundance likely reflects adequate propagule stores at the time of wildfire from mature standing plant species that can allocate more resources to reproduction as they increase in age and size (Smith et al., 2014; Strydom et al., 2017; Wenk & Falster, 2015). This demonstrates the resistance of these species to wildfire in older successional stages.

**FIGURE 7:**
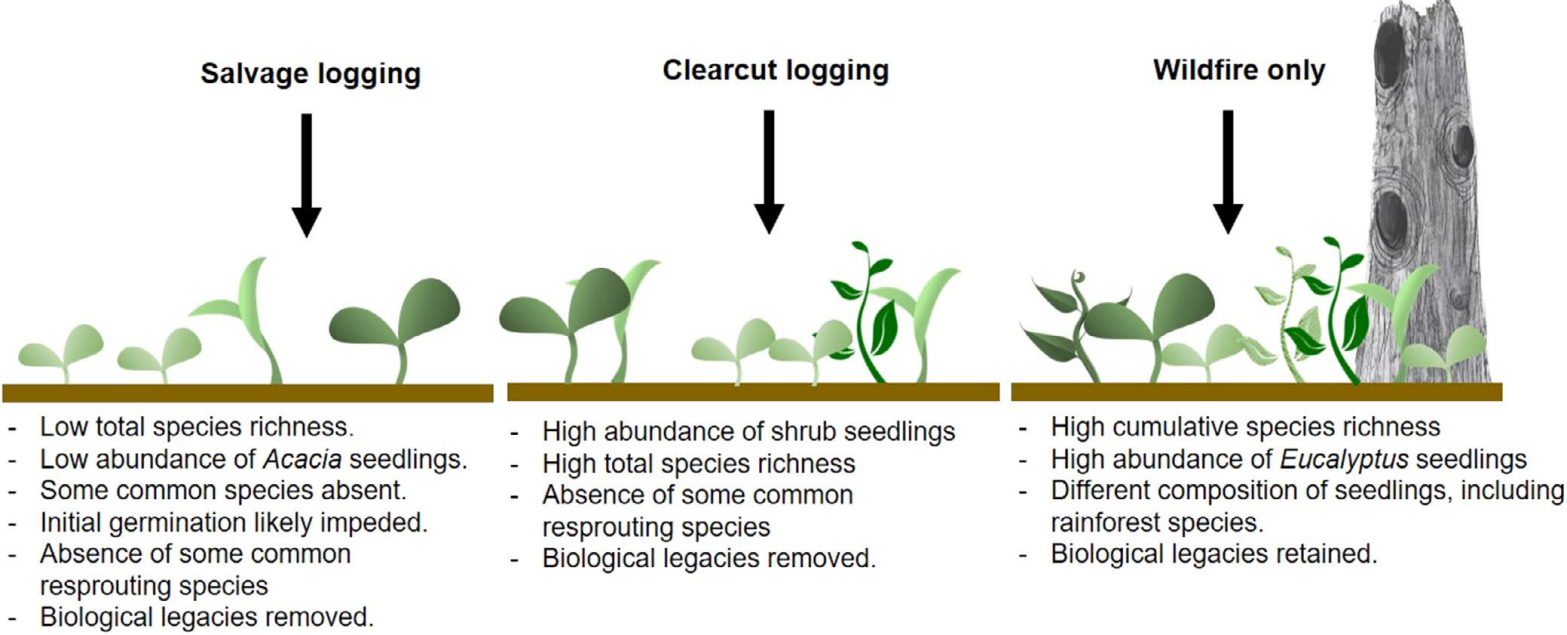
The influence of different disturbance histories on patterns of emerging seedlings disturbed in early‐successional (aged between 2007 and 2011) forests based on our major findings

Salvage logging after wildfire occurs in numerous forest ecosystems globally. It also occurs after pathogen attack, insect outbreak, or windthrow (Leverkus, Lindenmayer, et al., 2018; Thorn et al., 2018). Although common, this practice is highly controversial because it can have long‐lasting negative ecological consequences that impede forest recovery and resilience to future disturbances (Buma, 2015; Donato et al., 2006; Leverkus, Rey Benayas, et al., 2018; Lindenmayer & Noss, 2006; Seidl & Rammer, 2017; Taeroe et al., 2019). Consistent with prediction #3, we provide evidence that salvage logging can have a greater impact on forest regeneration, relative to forests with different disturbance histories of the same age. Specifically, we found the diversity of total seedlings and shrub seedlings emerging in salvage‐logged forests was the lowest relative to forests with different disturbance histories. They also supported the lowest number of regenerating *Acacia* species (Figure 6). Furthermore, the common tree species: *A*. *obliquinervia, A. frigescens, C. arcuealta, O. argophylla, P. axiflora, P. sambucifolia,* and *P. melissifolia* were absent from all salvage‐logged plots across the surveyed years; however, they occurred in forests of the same age with different disturbance histories (Table A6). The diversity of emerging seedlings also was low in other highly disturbed forests subject to compounding disturbances of two wildfires (1939 and 2009) and clearcut logging in 1970–90 (“Intermediate/2009F”). Specifically, these sites contained no evidence of the emergence of common shrub species *C. aculeata, P. axiflora, Z. arborescens, C. quadrifida* or *O. phlogopappa* across all sites. Similarly, in other forest ecosystems worldwide, salvage logging has been found to influence the structure, abundance, richness, and composition of regenerating plant communities (D'Amato et al., 2011; Donato et al., 2006; Leverkus et al., 2014; Parro et al., 2015; Sass et al., 2018). The influence of salvage logging on plant communities may be explained by the compounding influence of multiple disturbances, which can alter environmental conditions, exhaust reproductive propagules, and impede natural regeneration that may have occurred after initial natural disturbances (Leverkus, Lindenmayer, et al., 2018; Lindenmayer & Noss, 2006) (Figure 7).

The influence of disturbance history on emerging seedlings likely reflects the difference between wildfire and logging disturbances which can select for particular species and is consistent with biological/disturbance legacy successional theories (Blair et al., 2016; Franklin et al., 2000; Leverkus et al., 2014; Palik & Kastendick, 2009). For instance, wildfires produce long‐lasting biological legacies including dead and live standing trees which can increase structural heterogeneity and the proportion of emerging seedlings (Foster et al., 1998; Leverkus, et al., 2018). In contrast, logging operations involve mechanical disturbances that remove biological legacies (Lindenmayer & McCarthy, 2002), compact soils (Cambi et al., 2015), reduce the availability of soil nutrients (Bowd et al., 2019; Kishchuk et al., 2015), and kill resprouting structures (Blair et al., 2017; Bowd et al., 2018; Ough & Murphy, 2004)(Figure 7). For instance, in our study, common resprouting shrubs *O. argophylla* and *S. gaudichaudiana* were absent from sites clearcut and salvage logged in 2007–11, but occurred in all other unlogged, early‐successional sites. However, clearcut logging resulted in a high abundance and richness of other on‐site seeder shrub species including *C. aculeata, Ziera arborescens,* and *P. sambucifolia*.

While common species absent from some areas subject to disturbance histories may establish in later successional stages, patterns of seedling composition and density within the first five years postdisturbance can predict stand dynamics long‐term (Johnstone et al., 2004). Therefore, our observations may provide important insights into future stand development. However, we did not measure the proximity to source populations of mature trees, or prior populations which can also influence the richness, composition, and abundance of regeneration plant seedlings (Palmer et al., 2018; Primack & Miao, 1992; Tautenhahn et al., 2016). Further, we did not monitor seedling survival or growth rates, which require future research to support our findings.

### 4.3 Environmental influence on patterns of seedling emergence

Contrary to our fourth prediction at the outset of this investigation, we found a paucity of environmental influence on the abundance and composition of seedlings. However, our results show that higher indices of topographical wetness increased the probability of zero shrub seedlings, and influenced the composition of seedlings across all stand ages. Moreover, we found that early‐successional sites on a northerly aspect were characterized by a high abundance of shrub seedlings, but a low abundance of *Eucalyptus* seedlings. These results are likely attributed to species‐specific environmental preferences for germination (Ashton & Kelliher, 1996; Bell, 1994; Harper et al., 1965; Titus & del Moral, 1998). However, they also may reflect unmeasured variables associated with indices of topographical wetness and northerly aspects, such as temperature, solar radiation, and the density of surrounding vegetation (Aguilera et al., 2015; Ashton & Kelliher, 1996; Petter et al., 2015). The limited influence of environmental variables on seedlings suggests that disturbance history, stand age, and time since disturbance are the main drivers of patterns of seedling emergence in our study area.

## 5 CONCLUSIONS

Understanding seedling emergence after‐disturbance is important for predicting long‐term responses to future altered disturbance regimes in forests (Bače et al., 2015; Palmer et al., 2018; Sass et al., 2018). Our results provide evidence that stand age and disturbance history can influence the richness, composition and abundance of emerging seedlings. Specifically, persistent seedling emergence in older successional forests provides evidence of a mixed age understory, which may contribute to the resilience and persistence of some plant species over time in the event of future disturbances.

Our study also contributes to a growing body of literature which documents the negative influence of compounding disturbances such as salvage logging on recovering forest ecosystems (Leverkus, Lindenmayer, et al., 2018; Taeroe et al., 2019; Thorn et al., 2018). These disturbances can have major impacts in forests and produce successional trajectories that deviate from those shaped by natural disturbance (Lindenmayer & Noss, 2006; Paine et al., 1998; Taeroe et al., 2019). Moreover, compounding disturbances and interactions between individual disturbances can influence the resilience of ecosystems to future disturbances (Buma, 2015; Donato et al., 2006; Seidl & Rammer, 2017). Predicted increases in wildfires and other stand‐replacing natural disturbances (Abatzoglou & Williams, 2016; Bradstock et al., 2009; Jolly et al., 2015; Schoennagel et al., 2017) will likely result in an increase in prevalence of subsequent salvage logging operations (Leverkus, Lindenmayer, et al., 2018; Lindenmayer & Noss, 2006; Lindenmayer et al., 2017). Therefore, our findings provide a timely insight into the recovery of forests after salvage logging, relative to other disturbance histories. Specifically, our study highlights the importance of limiting anthropogenic perturbations, especially salvage logging, which may erode long‐term plant diversity, and undermine the resilience of plant communities in the event of additional future disturbances.

## CONFLICT OF INTEREST

The authors declare no conflict of interest.

## AUTHOR CONTRIBUTIONS

**Elle J. Bowd:** Data curation (supporting); Formal analysis (lead); Writing‐original draft (lead); Writing‐review & editing (lead). **Lachlan McBurney:** Data curation (equal); Project administration (equal); Resources (equal); Writing‐original draft (supporting). **David P. Blair:** Conceptualization (lead); Data curation (equal); Methodology (lead); Project administration (equal). **David B. Lindenmayer:** Conceptualization (equal); Funding acquisition (lead); Methodology (equal); Project administration (lead); Resources (equal); Supervision (lead); Writing‐original draft (equal).

## Figures and Tables

**TABLE A4:**
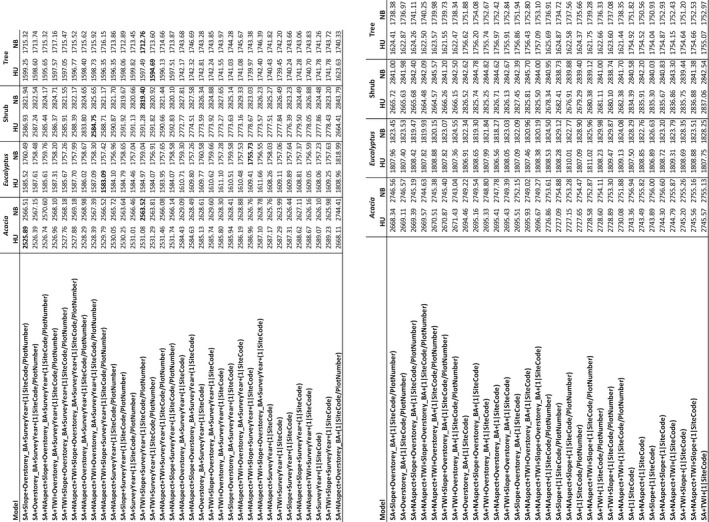
(a) Model Selection results for the Bayesian hurdle negative binomial regression models for shrub, *Eucalyptus, Acacia,* and tree lifeforms across all stand ages. (b) elpd_loo estimates and standard errors for the final model of each lifeform

Note

The values in the table are the WAIC (widely applicable information criteria). HU = hurdle model component, NB = negative binomial model component. Bold indicates the selected model with the lowest WAIC. SA = Stand age, Overstory BA = overstory of corresponding lifeform, NAspect = Northerly aspect.

**TABLE A8:**
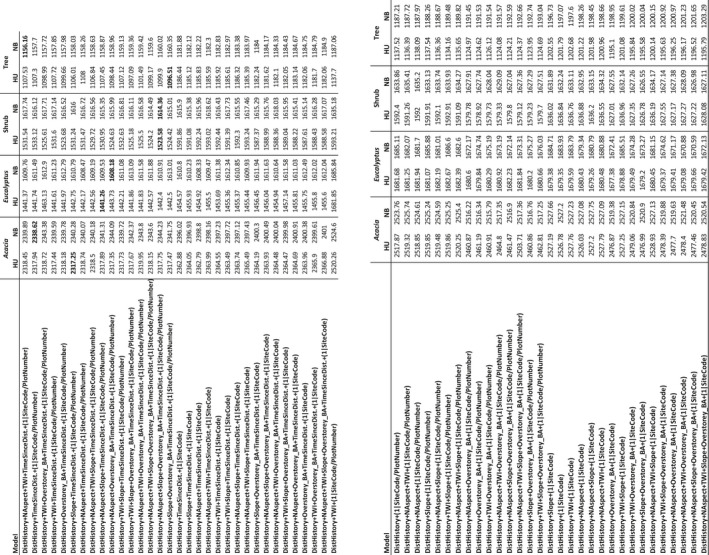
(a) Model Selection results for the Bayesian hurdle negative binomial regression models for shrub, *Eucalyptus, Acacia* and tree lifeforms in early‐successional forests. (b) elpd_loo estimates and standard errors for the final model of each lifeform

Note

The values in the table are the WAIC (widely applicable information criteria). HU = hurdle model component, NB = negative binomial model component. Bold indicates the selected model with the lowest WAIC. Overstory BA = overstory of corresponding lifeform, NAspect = Northerly aspect.

**FIGURE A4:**
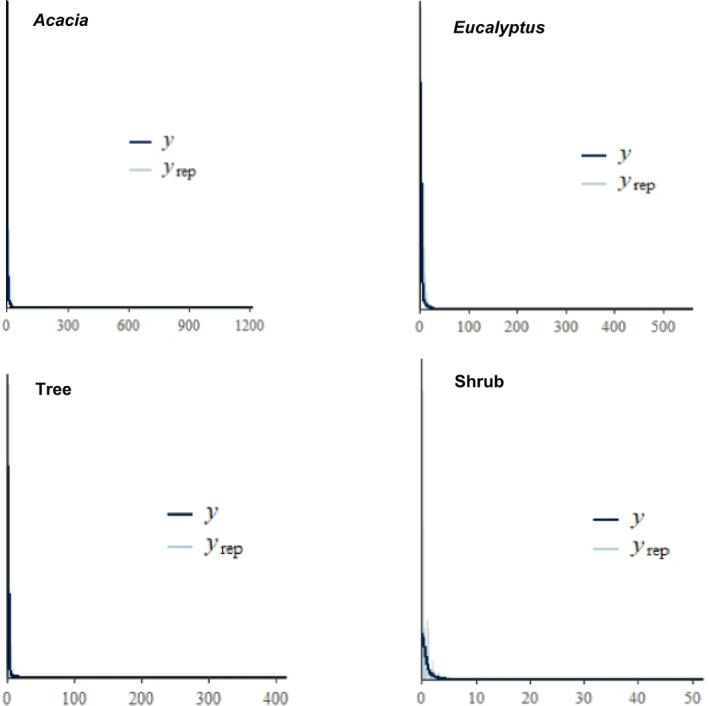
Post‐posterior predictive checks of each lifeform model across all stand ages. Plots display the kernel density estimates of the response generated from the posterior predictive distribution (*y*_rep_) against the distribution of the observed response (*y*)

**FIGURE A8:**
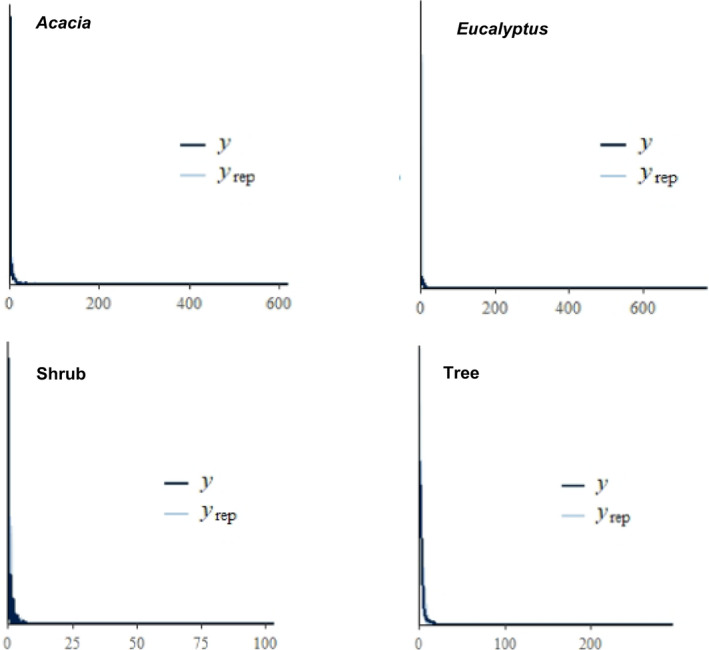
Post‐posterior predictive checks of each lifeform model in early successional forests. Plots display the kernel density estimates of the response generated from the posterior predictive distribution (*y*_rep_) against the distribution of the observed response (*y*)

## Data Availability

Data underpinning this study is accessible from Dryad via https://doi.org/10.5061/dryad.0cfxpnw1n.

## APPENDIX A

**TABLE A1:** Total no. of seedlings of each *Acacia, Eucalyptus*, tree, and shrub species pooled across all sites

Lifeform	Species	Total no. seedlings
*Acacia*	*Acacia dealbata*	1,431
*Acacia*	*Acacia obliquinervia*	1,078
*Acacia*	*Acacia frigescens*	531
*Acacia*	*Acacia nanodealbata*	60
*Acacia*	*Acacia* sp.	33
*Acacia*	*Acacia verticillata*	2
*Acacia*	*Acacia melanoxylon*	1
*Eucalyptus*	*Eucalyptus regnans*	3,374
*Eucalyptus*	*Eucalyptus delegatensis*	306
*Eucalyptus*	*Eucalyptus* sp.	162
*Eucalyptus*	*Eucalyptus nitens*	20
Shrub	*Polyscias sambucifolia*	366
Shrub	*Correa lawrenceana*	181
Shrub	*Prostanthera melissifolia*	169
Shrub	*Zieria arborescens*	86
Shrub	*Olearia phlogopappa*	81
Shrub	*Coprosma quadrifida*	63
Shrub	*Coprosma hirtella*	53
Shrub	*Pimelea axiflora*	47
Shrub	*Pultenaea muelleri*	38
Shrub	*Correa* sp.	27
Shrub	*Sambucus gaudichaudiana*	19
Shrub	*Cassinia aculeata*	17
Shrub	*Leucopogon gelidus*	12
Shrub	*Olearia argophylla*	12
Shrub	*Leionema bilobum*	10
Shrub	*Platylobium formosum*	10
Shrub	*Goodia lotifolia*	9
Shrub	*Daviesia mimosoides*	8
Shrub	*Philotheca myoporoides*	8
Shrub	*Daviesia latifolia*	7
Shrub	*Olearia lirata*	6
Shrub	*Solanum aviculare*	5
Shrub	*Pimelea linifolia*	4
Shrub	*Leptospermum* sp.	3
Shrub	*Acrothamnus maccraei*	2
Shrub	*Olearia* sp.	1
Shrub	*Ozothamnus thyrsoideus*	1
Shrub	*Pimelea ligustrina*	1
Tree	*Pomaderris aspera*	1,283
Tree	*Prostanthera lasianthos*	110
Tree	*Tasmannia lanceolata*	58
Tree	*Lomatia fraseri*	17
Tree	*Notelaea ligustrina*	15
Tree	*Nothofagus cunninghamii*	10
Tree	*Persoonia arborea*	5
Tree	*Pittosporum bicolor*	4
Tree	*Atherosperma moschatum*	2
Tree	*Hedycarya angustifolia*	2

**TABLE A2:** Grand mean abundance ± *SE* of each seedling for each stand age

Lifeform	Species	OG	Mature	Intermediate	Young
*Acacia*	*Acacia dealbata*	0.09 ± 0.038	0.009 ± 0.004	0.042 ± 0.025	1.994 ± 0.347
*Acacia*	*Acacia frigescens*	0.01 ± 0.01	0.003 ± 0.003		0.746 ± 0.208
*Acacia*	*Acacia melanoxylon*				0.001 ± 0.001
*Acacia*	*Acacia nanodealbata*		0.039 ± 0.032		0.049 ± 0.016
*Acacia*	*Acacia obliquinervia*				1.523 ± 0.412
*Acacia*	*Acacia* sp.		0.003 ± 0.003		0.044 ± 0.021
*Acacia*	*Acacia verticillata*				0.003 ± 0.002
*Eucalyptus*	*Eucalyptus delegatensis*		0.006 ± 0.003		0.427 ± 0.141
*Eucalyptus*	*Eucalyptus nitens*				0.028 ± 0.023
*Eucalyptus*	*Eucalyptus regnans*	0.01 ± 0.01	0.025 ± 0.012		4.742 ± 2.391
*Eucalyptus*	*Eucalyptus* sp.				0.229 ± 0.109
Shrub	*Acrothamnus maccraei*		0.003 ± 0.003		
Shrub	*Cassinia aculeata*		0.002 ± 0.002		0.023 ± 0.011
Shrub	*Coprosma hirtella*		0.04 ± 0.018		0.038 ± 0.018
Shrub	*Coprosma quadrifida*		0.019 ± 0.008		0.072 ± 0.017
Shrub	*Correa lawrenceana*	0.04 ± 0.02	0.113 ± 0.037	0.188 ± 0.068	0.121 ± 0.023
Shrub	*Correa* sp.		0.042 ± 0.033		
Shrub	*Daviesia latifolia*		0.011 ± 0.008		
Shrub	*Daviesia mimosoides*				0.011 ± 0.008
Shrub	*Goodia lotifolia*				0.013 ± 0.006
Shrub	*Leionema bilobum*		0.008 ± 0.005		0.007 ± 0.006
Shrub	*Leptospermum* sp.				0.004 ± 0.004
Shrub	*Leucopogon gelidus*		0.019 ± 0.014		
Shrub	*Olearia argophylla*		0.008 ± 0.006		0.01 ± 0.006
Shrub	*Olearia lirata*		0.005 ± 0.003		0.004 ± 0.003
Shrub	*Olearia phlogopappa*		0.014 ± 0.005	0.01 ± 0.01	0.1 ± 0.024
Shrub	*Olearia* sp.		0.002 ± 0.002		
Shrub	*Ozothamnus thyrsoideus*		0.002 ± 0.002		
Shrub	*Philotheca myoporoides*		0.012 ± 0.01		
Shrub	*Pimelea axiflora*		0.034 ± 0.011		0.035 ± 0.014
Shrub	*Pimelea ligustrina*				0.001 ± 0.001
Shrub	*Pimelea linifolia*				0.006 ± 0.006
Shrub	*Platylobium formosum*		0.015 ± 0.011		
Shrub	*Polyscias sambucifolia*	0.01 ± 0.01	0.179 ± 0.026	0.156 ± 0.06	0.331 ± 0.045
Shrub	*Prostanthera melissifolia*		0.022 ± 0.007		0.219 ± 0.091
Shrub	*Pultenaea muelleri*		0.059 ± 0.021		
Shrub	*Sambucus gaudichaudiana*		0.006 ± 0.003		0.021 ± 0.008
Shrub	*Solanum aviculare*				0.007 ± 0.004
Shrub	*Zieria arborescens*		0.019 ± 0.01	0.01 ± 0.01	0.103 ± 0.029
Tree	*Atherosperma moschatum*	0.02 ± 0.014			
Tree	*Hedycarya angustifolia*		0.002 ± 0.002		0.001 ± 0.001
Tree	*Lomatia fraseri*		0.015 ± 0.009		0.01 ± 0.005
Tree	*Notelaea ligustrina*		0.023 ± 0.008		
Tree	*Nothofagus cunninghamii*	0.04 ± 0.02	0.009 ± 0.007		
Tree	*Persoonia arborea*		0.002 ± 0.002		0.006 ± 0.006
Tree	*Pittosporum bicolor*		0.003 ± 0.002	0.021 ± 0.015	
Tree	*Pomaderris aspera*		0.002 ± 0.002		1.811 ± 0.363
Tree	*Prostanthera lasianthos*		0.039 ± 0.023		0.12 ± 0.026
Tree	*Tasmannia lanceolata*		0.082 ± 0.021	0.021 ± 0.021	0.004 ± 0.003

**TABLE A3:** Hurdle (hu) negative binomial (nb) posterior model summaries for the count of *Acacia*, *Eucalyptus*, tree, and shrub seedlings across all stand ages

	Estimate	l−95% CI	u−95% CI
No. of Tree seedlings			
nb_Intercept	−1.52	−3.45	0.27
nb_Mature SA	0.49	−1.41	2.48
nb_Intermediate SA	0.05	−2.54	2.51
nb_Young SA	2.27	**0.43**	**4.24**
nb_TWI (scaled)	0.3	−0.22	0.8
nb_Slope (scaled)	−0.1	−0.57	0.37
nb_Survey Year (scaled)	−0.68	**−1.03**	**−0.32**
hu_Intercept	4.52	2.69	6.43
hu_Mature SA	0.42	−1.49	2.34
hu_Intermediate SA	0.83	−1.64	3.48
hu_Young SA	−1.99	**−3.96**	**−0.05**
hu_TWI (scaled)	−0.37	−1.1	0.37
hu_Survey Year (scaled)	0.54	**0.31**	**0.79**
No. of *Acacia* seedlings			
nb_Intercept	−1.23	−2.88	0.29
nb_Mature SA	0.97	−0.86	2.89
nb_Intermediate SA	−0.49	−3.03	1.83
nb_Young SA	1.45	−0.13	3.1
nb_TWI (scaled)	−0.1	−0.57	0.35
nb_Slope (scaled)	−0.02	−0.48	0.45
nb_Survey Year (scaled)	−1.46	**−1.68**	**−1.24**
hu_Intercept	4.49	2.94	6.16
hu_Mature SA	1.96	**0.22**	**3.73**
hu_Intermediate SA	0.68	−1.34	2.95
hu_Young SA	−3.85	**−5.63**	**−2.17**
hu_Slope (scaled)	−0.01	−0.45	0.44
hu_Acacia BA (scaled)	0.27	−0.03	0.58
hu_Survey Year (scaled)	1.37	**1.09**	**1.67**
No. of *Eucalyptus* seedlings			
nb_Intercept	−0.89	−2.59	0.73
nb_Mature SA	0.7	−1.22	2.71
nb_Intermediate SA	−0.35	−3	2.18
nb_Young SA	1.31	−0.32	3.04
nb_Northerly aspect	−0.77	−1.77	0.2
nb_TWI (scaled)	−0.07	−0.55	0.37
nb_Slope (scaled)	0.02	−0.45	0.5
nb_Survey Year (scaled)	−1.47	**−1.75**	**−1.22**
hu_Intercept	4.29	2.66	6.02
hu_Mature SA	2.12	**0.4**	**3.98**
hu_Intermediate SA	0.89	−1.11	3.04
hu_Young SA	−3.83	**−5.6**	**−2.15**
hu_northerly aspect	0.42	−0.46	1.36
hu_TWI (scaled)	0.04	−0.43	0.5
hu_Slope (scaled)	0.02	−0.43	0.47
hu_Survey Year (scaled)	1.47	**1.19**	**1.76**
No. of Shrub seedlings			
nb_Intercept	−0.72	−2.31	0.77
nb_Mature SA	0.64	−0.85	2.19
nb_Intermediate SA	−0.08	−1.77	1.56
nb_Young SA	0.93	−0.55	2.48
nb_TWI (scaled)	−0.14	−0.43	0.14
nb_Slope (scaled)	−0.28	−0.59	0.01
nb_Survey Year (scaled)	−0.42	**−0.64**	**−0.22**
hu_Intercept	3.82	2.32	5.41
hu_Mature SA	−1.25	−2.88	0.31
hu_Intermediate SA	−1.25	−3.25	0.66
hu_Young SA	−2.36	**−4**	**−0.83**
hu_Northerly aspect	−0.43	−1.25	0.38
hu_TWI (scaled)	0.53	**0.11**	**0.94**
hu_Shrub BA (scaled)	0.36	**0.06**	**0.7**
hu_Survey Year (scaled)	0.72	**0.53**	**0.91**

Note

The regression parameters are on the log scale for conditional component of the model (negative binomial), and on the logit scale for the hurdle component of the model. The hurdle component is modeling the probability of a zero. Bold coefficients indicate significant associations. SA = stand age. The intercept represents old‐growth forests. See Table A4 for details of model selection and Figure A4 for diagnostic plots of the final model.

**TABLE A5:** (a) PERMANOVA analysis output, indicating the influence of stand age, TWI, slope, northerly aspect and the mean BA of *Eucalyptus*, tree, shrub and *Acacia* lifeforms on the composition of plant seedlings

	*Df*	SumsOfSqs	MeanSqs	*F*	*R* ^2^	*p*
Stand age	3	6.76	2.25	11.68	0.26	**0.001** ***
Slope	1	0.18	0.18	0.94	0.01	0.43
TWI	1	0.46	0.46	2.36	0.02	**0.028** *
Aspect	1	0.22	0.22	1.15	0.01	0.27
*Eucalyptus* BA (mean)	1	0.33	0.33	1.72	0.01	0.08
Shrub BA (mean)	1	0.21	0.21	1.09	0.01	0.34
Tree BA (mean	1	0.29	0.29	1.48	0.01	0.15
*Acacia* BA (mean)	1	0.26	0.26	1.37	0.01	0.18
Residuals	90	17.37	0.19		0.67	
Total	100	26.09			1.00	

(b) Pairwise comparisons of different stand ages, based on the composition of plant seedlings generated using PERMANOVA analysis					
Comparison	*Df*	SumsOfSqs	*F*	*R* ^2^	*p* (adjust.)
Mature versus Young	1	4.29	12.51	0.12	**0.006** **
Intermediate versus Young	1	1.65	5.64	0.09	**0.006** **
Young versus Old‐growth	1	1.03	3.45	0.06	**0.006** **
Mature versus Old‐growth	1	0.82	2.02	0.05	0.054
Intermediate versus Old‐growth	1	0.77	2.66	0.28	0.234
Mature versus Intermediate	1	0.52	1.32	0.03	1.000

Note

*p* values were adjusted using the Bonferroni method.

* *p* < 0.05

** *p* < 0.01

*** *p* < 0.001.

**TABLE A6:** Grand mean abundance ± *SE* of each seedling for each disturbance history for the first three years post‐disturbance

Lifeform	Species	OG/2009F	Mature/2009F	Intermediate/2009F
*Acacia*	*Acacia dealbata*	4.711 ± 1.132	1.285 ± 0.314	6.667 ± 3.148
*Acacia*	*Acacia frigescens*	0.011 ± 0.011	1.106 ± 0.388	
*Acacia*	*Acacia nanodealbata*	0.1 ± 0.079	0.049 ± 0.025	
*Acacia*	*Acacia obliquinervia*	7.211 ± 2.906	0.78 ± 0.294	0.939 ± 0.27
*Acacia*	*Acacia* sp.		0.187 ± 0.11	0.121 ± 0.095
*Acacia*	*Acacia verticillata*			
*Eucalyptus*	*Eucalyptus delegatensis*	0.556 ± 0.217	1.553 ± 0.767	0.273 ± 0.109
*Eucalyptus*	*Eucalyptus nitens*		0.163 ± 0.132	
*Eucalyptus*	*Eucalyptus regnans*	28.033 ± 18.478	2.496 ± 0.75	1.788 ± 0.537
*Eucalyptus*	*Eucalyptus* sp.		0.967 ± 0.533	0.652 ± 0.607
Shrub	*Cassinia aculeata*		0.016 ± 0.011	
Shrub	*Coprosma hirtella*	0.233 ± 0.128	0.008 ± 0.008	
Shrub	*Coprosma quadrifida*		0.146 ± 0.057	
Shrub	*Correa lawrenceana*	0.033 ± 0.025	0.163 ± 0.061	0.136 ± 0.048
Shrub	*Daviesia mimosoides*	0.089 ± 0.06		
Shrub	*Goodia lotifolia*		0.016 ± 0.016	0.015 ± 0.015
Shrub	*Leionema bilobum*			
Shrub	*Olearia argophylla*	0.022 ± 0.016		0.061 ± 0.061
Shrub	*Olearia lirata*			
Shrub	*Olearia phlogopappa*	0.122 ± 0.054	0.089 ± 0.04	
Shrub	*Pimelea axiflora*	0.189 ± 0.099		
Shrub	*Pimelea ligustrina*			
Shrub	*Polyscias sambucifolia*	0.333 ± 0.113	0.699 ± 0.152	
Shrub	*Prostanthera melissifolia*	0.056 ± 0.056		0.076 ± 0.054
Shrub	*Sambucus gaudichaudiana*	0.089 ± 0.044	0.024 ± 0.024	0.03 ± 0.021
Shrub	*Solanum aviculare*			
Shrub	*Zieria arborescens*	0.156 ± 0.052	0.049 ± 0.025	
Tree	*Hedycarya angustifolia*			
Tree	*Lomatia fraseri*		0.033 ± 0.023	
Tree	*Persoonia arborea*		0.033 ± 0.033	
Tree	*Pomaderris aspera*	2.156 ± 1.801	0.325 ± 0.12	0.061 ± 0.037
Tree	*Prostanthera lasianthos*	0.022 ± 0.022	0.187 ± 0.071	0.106 ± 0.049
Tree	*Tasmannia lanceolata*		0.024 ± 0.018	

Lifeform	Species	2007–11 CC	2009–11 SLV
*Acacia*	*Acacia dealbata*	3.333 ± 0.541	0.595 ± 0.174
*Acacia*	*Acacia frigescens*	3.065 ± 1.399	
*Acacia*	*Acacia nanodealbata*		
*Acacia*	*Acacia obliquinervia*	1.989 ± 1.098	
*Acacia*	*Acacia* sp.		
*Acacia*	*Acacia verticillata*		0.048 ± 0.033
*Eucalyptus*	*Eucalyptus delegatensis*	0.398 ± 0.219	
*Eucalyptus*	*Eucalyptus nitens*		
*Eucalyptus*	*Eucalyptus regnans*	3.731 ± 2.645	1.262 ± 0.279
*Eucalyptus*	*Eucalyptus* sp.		
Shrub	*Cassinia aculeata*	0.151 ± 0.085	
Shrub	*Coprosma hirtella*	0.054 ± 0.044	
Shrub	*Coprosma quadrifida*	0.097 ± 0.046	0.31 ± 0.165
Shrub	*Correa lawrenceana*	0.333 ± 0.121	0.19 ± 0.124
Shrub	*Daviesia mimosoides*		
Shrub	*Goodia lotifolia*		
Shrub	*Leionema bilobum*	0.043 ± 0.043	
Shrub	*Olearia argophylla*		
Shrub	*Olearia lirata*	0.032 ± 0.024	
Shrub	*Olearia phlogopappa*	0.419 ± 0.162	0.095 ± 0.057
Shrub	*Pimelea axiflora*	0.054 ± 0.035	
Shrub	*Pimelea ligustrina*		0.024 ± 0.024
Shrub	*Polyscias sambucifolia*	1.054 ± 0.224	
Shrub	*Prostanthera melissifolia*	1.548 ± 0.678	
Shrub	*Sambucus gaudichaudiana*		
Shrub	*Solanum aviculare*	0.022 ± 0.015	0.071 ± 0.053
Shrub	*Zieria arborescens*	0.43 ± 0.198	0.071 ± 0.053
Tree	*Hedycarya angustifolia*	0.011 ± 0.011	
Tree	*Lomatia fraseri*		
Tree	*Persoonia arborea*		
Tree	*Pomaderris aspera*	8.011 ± 1.877	3.595 ± 0.767
Tree	*Prostanthera lasianthos*	0.258 ± 0.131	0.381 ± 0.207
Tree	*Tasmannia lanceolata*		

**TABLE A7:** Hurdle (hu) truncated negative binomial (nb) posterior model summaries for the count of *Acacia, Eucalyptus*, tree and shrub seedlings in early successional forests with different disturbance histories

	Estimate	l−95% CI	u−95% CI
No. *Acacia* seedlings			
nb_Intercept	0.64	−0.22	1.45
nb_Mature/2009F	−0.78	−1.81	0.27
nb_Intermediate/2009F	−0.27	−1.46	0.99
nb_2007−11 CC	0.02	−0.96	1
nb_2009−11 SLV	−1.96	**−3.33**	**−0.65**
nb_Time since disturbance (scaled)	−1.54	**−1.78**	**−1.32**
hu_Intercept	0.86	−0.16	1.93
hu_Mature/2009F	0.05	−1.32	1.44
hu_Intermediate/2009F	−0.32	−1.99	1.24
hu_2007−11 CC	−1.13	−2.54	0.22
hu_2009−11 SLV	1.34	−0.22	2.93
hu_Slope (scaled)	−0.21	−0.7	0.29
hu_Time since disturbance (scaled)	1.71	**1.39**	**2.06**
No. *Eucalyptus* seedlings			
nb_Intercept	−0.85	−1.94	0.23
nb_Mature/2009F	−0.63	−1.76	0.53
nb_Intermediate/2009F	−1.02	−2.36	0.37
nb_2007−11 CC	−0.25	−1.36	0.98
nb_2009−11 SLV	0.19	−1.08	1.51
nb_AspectN	−1.98	**−3.17**	**−0.78**
nb_Eucalypt_BA (scaled)	−0.01	−0.25	0.25
nb_Time since disturbance (scaled)	−3.18	**−3.89**	**−2.44**
hu_Intercept	2.3	1.26	3.44
hu_Mature/2009F	0.53	−0.64	1.71
hu_Intermediate/2009F	0.42	−1.03	1.83
hu_2007−11 CC	0.91	−0.29	2.17
hu_2009−11 SLV	0.07	−1.31	1.44
hu_AspectN	0.7	−0.39	1.77
hu_Slope (scaled)	−0.06	−0.51	0.39
hu_Time since disturbance (scaled)	3.12	**2.53**	**3.83**
No. Shrub seedlings			
nb_Intercept	−0.9	−1.96	−0.12
nb_Mature/2009F	0.66	−0.12	1.46
nb_Intermediate/2009F	−0.44	−1.54	0.69
nb_2007−11 CC	1.55	**0.78**	**2.36**
nb_2009−11 SLV	0.07	−0.94	1.14
nb_AspectN	0.69	**0.05**	**1.29**
nb_Slope (scaled)	−0.18	−0.43	0.07
nb_Shrub_BA (scaled)	−0.41	−1.01	0.1
nb_Time since disturbance (scaled)	−0.66	**−1.02**	**−0.33**
hu_Intercept	1.35	0.34	2.41
hu_Mature/2009F	0.08	−1.13	1.24
hu_Intermediate/2009F	1.31	−0.2	2.95
hu_2007−11 CC	−0.77	−2.04	0.5
hu_2009−11 SLV	1.02	−0.43	2.5
hu_AspectN	−0.67	−1.73	0.41
hu_Slope (scaled)	−0.51	**−0.96**	**−0.08**
hu_Shrub_BA (scaled)	0.41	**0.01**	**0.92**
hu_Time since disturbance (scaled)	0.87	**0.61**	**1.14**
No. Tree seedlin			
nb_Intercept	0.66	−0.91	2.12
nb_Mature/2009F	−0.69	−2.21	0.88
nb_Intermediate/2009F	−1.24	−3	0.48
nb_2007−11 CC	0.93	−0.55	2.49
nb_2009−11 SLV	−0.72	−2.17	0.74
nb_AspectN	0.2	−0.91	1.29
nb_TWI (scaled)	0.09	−0.38	0.54
nb_Time since disturbance (scaled)	−0.99	**−1.41**	**−0.58**
hu_Intercept	3.36	1.56	5.33
hu_Mature/2009F	0.37	−1.83	2.78
hu_Intermediate/2009F	0.14	−2.32	2.69
hu_2007−11 CC	−1.72	−4.15	0.66
hu_2009−11 SLV	−2.23	−5.32	0.45
hu_Slope (scaled)	−0.08	−1.17	0.97
hu_Tree BA (scaled)	0.43	−0.04	0.96
hu_Time since disturbance (scaled)	0.88	**0.5**	**1.31**

Note

The regression parameters are on the log scale for conditional component of the model (truncated negative binomial), and on the logit scale for the hurdle component of the model. The hurdle component is modelling the probability of a zero. “CC” = clearcut logging, “F” = fire, “SLV” = salvage logging. Bold coefficients indicate a significant association. The intercept represents old‐growth forests, burnt in 2009. See Table A8 for details of model selection, and Figure A8 for diagnostic plots of the final model.

**TABLE A9:** (a) PERMANOVA analysis output, indicating the influence of disturbance history, TWI, slope, northerly aspect and the mean BA of *Eucalyptus*, tree, shrub and *Acacia* lifeforms on the composition of plant seedlings

	*Df*	SumsOfSqs	MeanSqs	*F*	*R* ^2^	*p*
Disturbance history	4	2.43	0.61	2.33	0.15	**0.00** ***
Slope	1	0.25	0.25	0.97	0.02	0.47
TWI	1	0.38	0.38	1.45	0.02	0.15
Aspect	1	0.44	0.44	1.70	0.03	0.09
*Eucalyptus* BA (mean)	1	0.49	0.49	1.88	0.03	**0.04** *
Shrub BA (mean)	1	0.19	0.19	0.72	0.01	0.74
Tree BA (mean	1	0.31	0.31	1.20	0.02	0.27
*Acacia* BA (mean)	1	0.43	0.43	1.66	0.03	0.09
Residuals	43	11.21	0.26		0.69	
Total	54	16.13			1.00	

(b) Pairwise comparisons of different disturbance histories, based on the composition of plant seedlings generated using PERMANOVA analysis. “CC” = clearcut logging, “F” = fire, “SLV” = salvage logging					
Comparison	*Df*	SumsOfSqs	*F*	*R* ^2^	*p* (adjust.)
2007–11 CC versus OG/2009F	1	0.41	1.45	0.06	1
2007–11 CC versus Mature/2009F	1	0.50	1.67	0.05	0.75
2007–11 CC versus Intermediate/2009F	1	0.72	2.75	0.11	**0.03** *
2007–11 CC versus 2009–11 SLV	1	0.89	3.70	0.12	**0.02** *
OG/2009F versus Mature/2009F	1	0.33	1.00	0.05	1
OG/2009F versus Intermediate/2009F	1	0.26	0.95	0.07	1
OG/2009F versus 2009–11 SLV	1	0.83	3.49	0.18	**0.02** *
Mature/2009F versus Intermediate/2009F	1	0.31	1.00	0.06	1
Mature/2009F versus 2009–11 SLV	1	1.00	3.62	0.15	**0.01** *
Intermediate/2009F versus 2009–11 SLV	1	0.69	3.44	0.20	0.07

Note

*p* values were adjusted using the bonferroni method.

* *p* < 0.05

** *p* < 0.01

*** *p* < 0.001.
